# The *Listeria monocytogenes* PASTA Kinase PrkA and Its Substrate YvcK Are Required for Cell Wall Homeostasis, Metabolism, and Virulence

**DOI:** 10.1371/journal.ppat.1006001

**Published:** 2016-11-02

**Authors:** Daniel A. Pensinger, Kyle M. Boldon, Grischa Y. Chen, William J. B. Vincent, Kyle Sherman, Meng Xiong, Adam J. Schaenzer, Emily R. Forster, Jörn Coers, Rob Striker, John-Demian Sauer

**Affiliations:** 1 Department of Medical Microbiology and Immunology University of Wisconsin-Madison, School of Medicine and Public Health, Madison, Wisconsin; 2 Department of Medicine, University of Wisconsin-Madison, School of Medicine and Public Health, Madison, Wisconsin; 3 Department of Molecular Genetics and Microbiology, Duke University School of Medicine, Durham, North Carolina; 4 W. S. Middleton Memorial Veteran’s Hospital, Madison, Wisconsin; University of Michigan Medical School, UNITED STATES

## Abstract

Obstacles to bacterial survival and replication in the cytosol of host cells, and the mechanisms used by bacterial pathogens to adapt to this niche are not well understood. *Listeria monocytogenes* is a well-studied Gram-positive foodborne pathogen that has evolved to invade and replicate within the host cell cytosol; yet the mechanisms by which it senses and responds to stress to survive in the cytosol are largely unknown. To assess the role of the *L*. *monocytogenes* penicillin-binding-protein and serine/threonine associated (PASTA) kinase PrkA in stress responses, cytosolic survival and virulence, we constructed a Δ*prkA* deletion mutant. PrkA was required for resistance to cell wall stress, growth on cytosolic carbon sources, intracellular replication, cytosolic survival, inflammasome avoidance and ultimately virulence in a murine model of Listeriosis. In *Bacillus subtilis* and *Mycobacterium tuberculosis*, homologues of PrkA phosphorylate a highly conserved protein of unknown function, YvcK. We found that, similar to PrkA, YvcK is also required for cell wall stress responses, metabolism of glycerol, cytosolic survival, inflammasome avoidance and virulence. We further demonstrate that similar to other organisms, YvcK is directly phosphorylated by PrkA, although the specific site(s) of phosphorylation are not highly conserved. Finally, analysis of phosphoablative and phosphomimetic mutants of YvcK *in vitro* and *in vivo* demonstrate that while phosphorylation of YvcK is irrelevant to metabolism and cell wall stress responses, surprisingly, a phosphomimetic, nonreversible negative charge of YvcK is detrimental to cytosolic survival and virulence *in vivo*. Taken together our data identify two novel virulence factors essential for cytosolic survival and virulence of *L*. *monocytogenes*. Furthermore, our data demonstrate that regulation of YvcK phosphorylation is tightly controlled and is critical for virulence. Finally, our data suggest that yet to be identified substrates of PrkA are essential for cytosolic survival and virulence of *L*. *monocytogenes* and illustrate the importance of studying protein phosphorylation in the context of infection.

## Author Summary

Infection with intracellular pathogens causes a majority of the global infectious disease associated mortality. A number of intracellular pathogens must directly access the host cytosol in order to cause disease; however, non-cytosol adapted bacteria do not survive or replicate upon access to the cytosol. The mechanisms cytosolic pathogens use to adapt to this niche are largely unknown. The model cytosolic bacterial pathogen *Listeria monocytogenes* contains a single penicillin-binding-protein and serine/threonine associated (PASTA) kinase, PrkA. In other bacteria, PASTA kinases bind cell wall fragments and phosphorylate downstream effectors involved in cell wall synthesis, central metabolism, virulence, cell division, and biofilm formation. We demonstrate that in *L*. *monocytogenes*, PrkA is required for cell wall homeostasis, growth under nutrient limiting conditions, survival and replication in host cells, and virulence *in vivo*. Furthermore, we identify a highly conserved protein of unknown function, YvcK, as a PrkA substrate. We demonstrate that *L*. *monocytogenes* YvcK is similarly required for cell wall stress responses, growth on glycerol, cytosolic survival and virulence *in vivo*. Surprisingly, a phosphomimetic, nonreversible negative charge at the phosphorylation sites on YvcK inactivates functions of the protein related to intracellular survival and virulence, suggesting that the identification of PASTA kinase substrates phosphorylated during infection will be critical to our understanding of this central regulator metabolism, cell wall homeostasis and ultimately virulence.

## Introduction

Intracellular pathogens are responsible for a majority of the world’s most devastating infectious disease. A subset of intracellular pathogens, known as cytosolic pathogens, survive and thrive in the host cell cytoplasm. Recently, it has become clear that some traditionally extracellular or vacuolar pathogens such as *Staphylococcus aureus* [1] and *Mycobacterium tuberculosis* [2], respectively, spend at least part of their life in the cytosol. For canonical cytosolic pathogens like *Listeria monocytogenes and Francisella tularensis*, mutants that either cannot access the cytosol [3,4] or fail to survive [5,6] and replicate [7,8] within the cytosol are avirulent. Importantly, the cytosol is restrictive to bacterial replication, as both non-pathogenic bacteria [9], and even some pathogens that normally localize to other cellular compartments [10], are unable to replicate when localized to the cytosol, suggesting that specific adaptions are required for cytosolic survival. While the stresses and their cognate bacterial responses present in the phagosome have been extensively studied, obstacles to bacterial survival and replication in the cytosol are relatively unexplored. Potential barriers to survival and replication in the cytosol include cell wall stresses [5], metabolic restriction [11] and direct antimicrobial effectors such as ubiquidicin [12] and Guanylate Binding Proteins (GBPs) [13,14]. The mechanisms cytosolic pathogens use to sense and respond to these obstacles in order to survive in this restrictive niche are also unknown.

In addition to avoiding direct antimicrobial activities of the host cell cytosol, evading detection by the innate immune system is another major challenge for cytosolic pathogens. The last decade has seen an explosion of knowledge about how host cells detect invading bacteria and viruses in the cytosol, and in some cases, pathogen strategies for avoiding detection [15,16]. Specifically, bacteriolysis in the cytosol triggers both cGAS/STING-mediated type I interferon responses and AIM2-dependent inflammasome activation [17,18]. In the case of AIM2, DNA released during bacterolysis directly binds to AIM2, leading to oligimerization with ASC and Caspase 1 to form a functional inflammasome complex [19]. Activation of the inflammasome leads to a robust inflammatory response and the loss of the intracellular replication niche through a lytic, inflammatory cell death called pyroptosis [20–22]. As such, avoiding bacteriolysis is crucial for evasion of the host immune response and the ultimate success of cytosolic pathogens [22].

*Listeria monocytogenes* is a gram-positive, facultative intracellular pathogen that lives as a saprophyte in the soil and is most commonly contracted through contaminated food products leading to systemic Listeriosis [23]. Listeriosis is a significant contributor to total fatalities caused by food borne illness in the United States and elsewhere, with a fatality rate of up to 30% even with antibiotic treatment [24]. *L*. *monocytogenes* is also an ideal model to study cytosolic pathogens and host responses as it is genetically tractable, has a well-defined intracellular lifecycle and has robust *ex vivo* and *in vivo* models to study pathogenesis [25,26]. After ingestion, *L*. *monocytogenes* is either phagocytosed by professional phagocytes or induces entry into epithelial cells by receptor mediated endocytosis [27,28]. To escape the vacuole the bacteria secrete the pore forming toxin Listeriolysin O (LLO) encoded by the gene *hly* [3]. Accessing the cytosol is crucial for *L*. *monocytogenes* to cause disease as mutants that lack *hly* are avirulent [3,29]. Once inside the cytoplasm the bacteria replicate to high numbers and hijack host actin to propel themselves into neighboring cells [30]. Upon spread to neighboring cells, *L*. *monocytogenes* again expresses *hly* and a pair of phospholipases to escape the secondary vacuole and continue its infectious lifecycle [31,32]. While the mechanisms used by *L*. *monocytogenes* to access the cytosol and spread from cell-to-cell are well defined, the mechanisms by which *L*. *monocytogenes* senses and adapts to the cytosolic environment are not well understood.

One way bacteria respond to their environment is through sensor kinases that reversibly phosphorylate effector proteins. Classically, bacterial signal transduction through phosphorylation has been thought to be primarily mediated through two-component systems[33]. However, it has recently become clear that in many Gram-positive bacteria the eukaryotic-like kinases such as the Penicillin-Binding Protein And Serine/Threonine Kinase-Associated Protein (PASTA) kinases phosphorylate proteins to regulate diverse cellular processes ranging from cell wall synthesis [34,35] and cell division [36], to central metabolism [37], biofilm formation [38], and virulence [39]. PASTA kinases are composed of extracellular penicillin-binding-protein domains which, upon binding of peptidoglycan fragments, facilitate dimerization of the intracellular kinase domains, autophosphorylation and ultimately phosphorylation of downstream effector proteins [40,41]. PknB, the PASTA kinase in *M*. *tuberculosis* is essential and has been investigated as a potential drug target [42–44], while the PASTA kinases in *Staphylococcus epidermidis* and *Streptococcus pyogenes* have been shown to regulate biofilm formation, production of virulence factors and cell wall homeostasis [38,45]. We previously demonstrated that the *L*. *monocytogenes* PASTA kinase, PrkA, is required for β-lactam resistance, however, its role in infection and virulence is unknown [46].

A variety of PASTA kinase substrates have been defined in different organisms. One conserved substrate in *M*. *tuberculosis* and *B*. *subtilis* is YvcK (also known as CuvA in *M*. *tuberculosis*), a highly conserved protein of unknown function [36,47]. In *M*. *tuberculosis* and *B*. *subtilis* YvcK is singly phosphorylated on a threonine residue near the C-terminus, but the site of phosphorylation is not precisely conserved [36,47]. Although the biochemical function of YvcK is unknown, like the PASTA kinases themselves, YvcK is important for both carbon metabolism and cell wall homeostasis [48–50]. In *B*. *subtilis* and *M*. *tuberculosis* YvcK is required for growth on gluconeogenic substrates, pentose phosphate pathway/citric acid cycle intermediates and cholesterol, respectively [48,50]. Additionally, both *B*. *subtilis* and *M*. *tuberculosis ΔyvcK* mutants display morphology defects under gluconeogenic growth conditions [48,50]. Although *M*. *tuberculosis* CuvA localizes to the division septum and the poles, whereas *B*. *subtilis* YvcK localizes in a helical pattern, in both organisms CuvA/YvcK is required for Pbp1 localization under gluconeogenic growth conditions [49,50]. Finally, in *B*. *subtilis*, YvcK mediated localization of Pbp1 was dependent on phosphorylation by the PASTA kinase, [47]. *L*. *monocytogenes* YvcK is required for cytosolic survival and evasion of the AIM2 inflammasome and Δ*yvcK* mutants are hypersusceptible to lysozyme *in vitro* [17,51]. The interaction of *L*. *monocytogenes* YvcK with PrkA, as well as its role in cell wall homeostasis, carbon metabolism or virulence are unknown [17].

In this study, we characterize the function of PrkA in cell wall homeostasis, metabolism, cellular infection and ultimately virulence. Similar to what was previously observed in *M*. *tuberculosis* and *B*. *subtilis*, PrkA is important for dealing with both metabolic and cell wall stress in *L*. *monocytogenes*. Furthermore, PrkA is required for intracellular replication, cytosolic survival, evasion of the AIM2 inflammasome and ultimately virulence in murine models of listeriosis. In addition, we find that the conserved PASTA kinase substrate YvcK is a PrkA substrate in *L*. *monocytogenes*, but that the sites of phosphorylation are significantly different from those previously described in other organisms. Furthermore, similar to Δ*prkA* mutants, Δ*yvcK* mutants are also sensitive to metabolic and cell wall stress and are required for cytosolic survival and ultimately virulence *in vivo*. Surprisingly however, despite the fact that both PrkA and its substrate YvcK are required for cytosolic survival and virulence, phosphomimetic negative charges at the phosphorylation sites on YvcK inhibit its functions during infection. These data suggest that spatial and temporal regulation of YvcK phosphorylation is critical, and further suggest that alternative PrkA substrates are required for cytosolic survival and ultimately virulence. Given the highly conserved nature of both PrkA and YvcK in a variety of high impact pathogens, as well as their essential role in virulence in both *M*. *tuberculosis* and *L*. *monocytogenes*, compounds that interfere with this signaling axis may represent a promising new approach to antibiotic development.

## Results

### PrkA is required for cell wall homeostasis and glycerol metabolism *in vitro*

We had previously demonstrated, similar to what has been observed for PASTA kinase mutants in other organisms, that the *L*. *monocytogenes* PASTA kinase PrkA was required for resistance to β-lactam antibiotics, but not vancomycin [46], suggesting that PrkA regulates specific steps in cell wall synthesis and/or remodeling. To further characterize the function of PrkA in cell wall homeostasis, we determined the MIC of wild-type and Δ*prkA* mutant *L*. *monocytogenes* to a variety of additional cell wall inhibitors (Table 1). Similar to what we had observed with β-lactams, we found that the Δ*prkA* mutant is highly susceptible to tunicamycin (~100-fold increased sensitivity), an inhibitor that prevents the attachment of peptidoglycan and wall teichoic acid precursors to their lipid carrier. Complementation of the Δ*prkA* mutant restores wild type levels of tunicamycin resistance (S1A Fig). Additionally the Δ*prkA* mutant was slightly more susceptible to Bacitracin and lysozyme, inhibitors that target undecaprenal recycling and the β-(1–4)-glycosidic bonds between N-acetylmuramic acid and N-acetylglucosamine in peptidoglycan, respectively. Increased sensitivity to the human antimicrobial peptide LL-37 suggests a role for PrkA in regulating teichoic acid biosynthesis/modification [52]. These data suggest that PrkA has a role in regulating multiple steps in cell wall synthesis and maintenance.

**Table 1:** Minimum inhibitory concentrations of cell wall targeting agents in BHI. Values are mean minimum inhibitory concentrations and standard deviations in μg/mL. Values were determined by five or more biological replicates of serial 2-fold dilutions up or down from 1 μg/mL. Shaded boxes are statistically significant compared to wild-type (Student’s T-Test P< 0.05).

	Wild-Type	*ΔprkA*	Δ*yvcK*
Ampicillin	0.188 ± **0.068**	0.014 ± **0.003**	0.104 ± **0.032**
Bacitracin	205 ± **70.1**	116 ± **28.6**	128 ± **0**
Ceftriaxone	4.86 ± **2.27**	0.037 ± **0.025**	1.93 ± **1.10**
LL-37	>128 ± **0**	8 ± **0**	50.7 ± **41**
Lysozyme	>4096 ± **0**	1195 ± **418**	1195 ± **418**
Tunicamycin	4 ± **0**	0.044 ± **0.017**	4 ± **0**
Vancomycin	3.33 ± **2.42**	1.67 ± **0.516**	2.83 ± **2.56**

**Fig 1:**
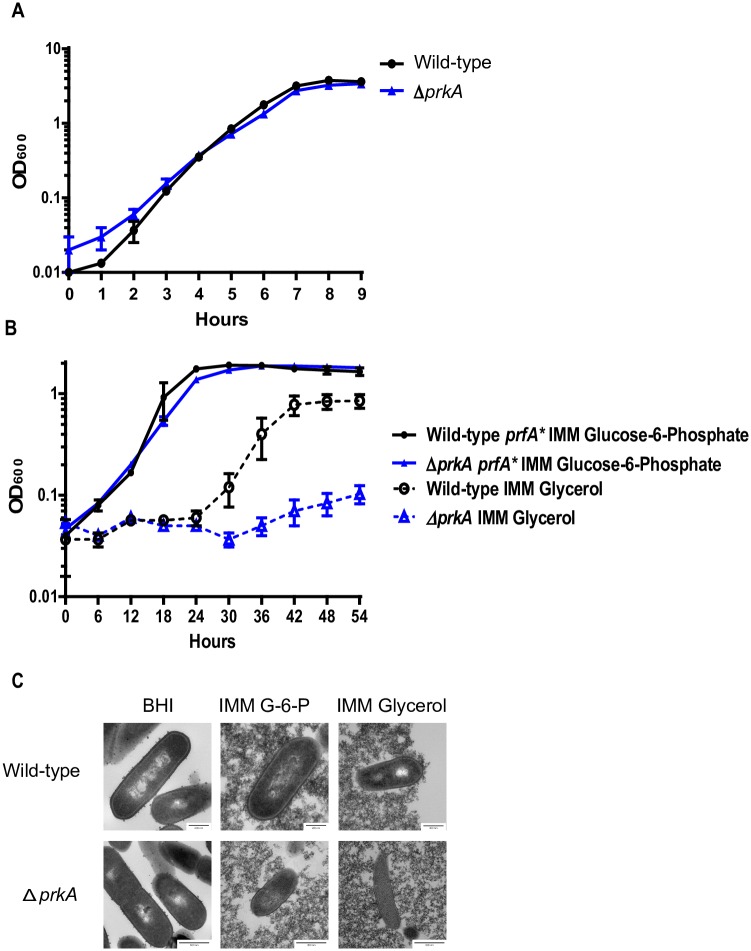
The Δ*prkA* mutant has altered growth and morphology in minimal media with glycerol as the primary carbon source. **(A)** Growth of the Δ*prkA* mutant in BHI, **(B)** or in Improved Minimal Media (IMM) with glucose-6-phosphate (filled symbols and solid lines) or glycerol (open symbols and dashed lines) as the sole carbon source. Data are averages of three biological replicates and error bars represent standard deviation of the mean. **(C)** Morphology of wild-type and the Δ*prkA* mutant in BHI, IMM Glucose-6-phosphate, and IMM Glycerol at OD 0.5 (BHI) or 6 hours post inoculation.

In many organisms, PASTA kinases phosphorylate central metabolic enzymes. Glycerol and phosphorylated glucose are the two primary carbon sources used by *L*. *monocytogenes* during intracellular growth [53,54]. Therefore, to assess potential metabolic deficiencies in Δ*prkA* mutants that could be relevant to virulence, we assayed growth of wild type and ΔprkA mutants in minimal media with either glucose-6-phosphate or glycerol as the primary carbon sources. As *L*. *monocytogenes* only expresses the glucose-6-phosphate transporter (*hpt*) when the master virulence regulator PrfA is active we used strains with constitutively active PrfA (prfA*) to assess growth on glucose-6-phosphate [11]. There was no detectable difference in growth between wild type and Δ*prkA* mutants in rich media (Fig 1A), or in Improved Minimal Media (IMM) with glucose-6-phosphate (Fig 1B). Importantly, despite increased sensitivity to cell wall acting antibiotics, there were no defects in bacterial morphology (Fig 1C, S1 Fig) or cell wall thickness (24.2 ± 1.43nm for wild-type vs 24.9 ±2.47 nm for Δ*prkA*) in rich media or minimal media with glucose-6-phosphate. Conversely, Δ*prkA* mutants were essentially unable to replicate in minimal media with glycerol as the sole carbon source with a doubling time of 16.3 hours compared to 4.5 hours for wild-type (Fig 1B). Complementation of *prkA* expression *in trans* restored growth in minimal media with glycerol (S2B Fig). Consistent with a pleiotropic role in both carbon metabolism and cell wall homeostasis, Δ*prkA* mutants displayed morphological defects in minimal media with glycerol (Fig 1C, S2 Fig). Taken together, these data suggest that, similar to its role in other organisms, the PASTA kinase PrkA is required for cell wall homeostasis and central metabolism in *L*. *monocytogenes*.

### PrkA is required for *L*. *monocytogenes* intracellular replication, cytosolic survival, avoidance of the AIM2 inflammasome and ultimately virulence

**Fig 2:**
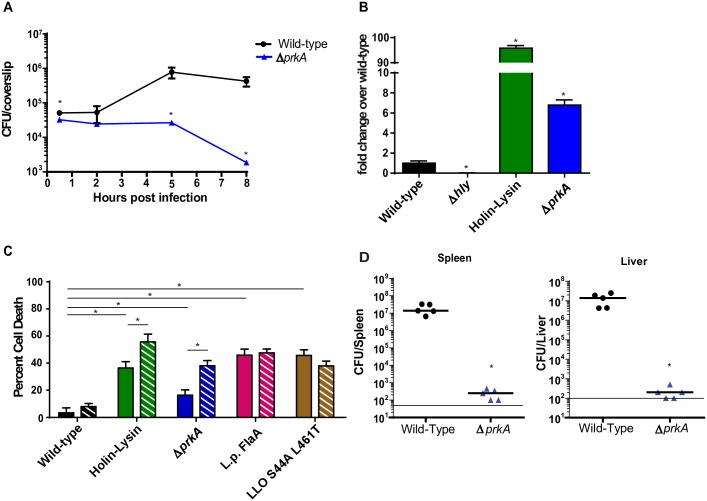
PrkA is required for intracellular replication, cytosolic survival, evasion of inflammasome activation and virulence. **(A)** Intracellular growth of wild-type (black circles) and Δ*prkA* mutants (blue triangles) was determined in bone marrow-derived macrophages (BMDMs) following infection at an MOI of 0.2. **(B)** Intracellular lysis of *L*. *monocytogenes* was measured in immortalized interferon α/β receptor (IFNAR)^-/-^ macrophages. Macrophages were infected at an MOI of 5 by *L*. *monocytogenes* strains carrying pBHE573 and macrophage luciferase expression was measured 6 hours post infection. **(A, B)** Mean values are reported. Experiments are representative of three or more biological replicates and error bars represent the standard deviation of technical triplicates. * indicates statistical significance by Student’s T-test (P<0.05). **(C)** Host Cell Death induced in wild-type C57/Bl6 (solid) or Gbp^Chr3-/-^ (hashed) BMDMs was analyzed by lactate dehydrogenase release 6 hours post infection at an MOI of 5. Data is an average of three biological replicates and error bars represent standard deviation of the mean. * indicates statistical significance by Student’s T-test (P<0.05). **(D)** C57Bl6 mice were infected intravenously with 1x10^5^ wild-type (black circles) or Δ*prkA* mutants (blue triangles) *in vivo*. Spleens (left) and Livers (right) were harvested 48 hours post infection homogenized and plated for CFU. The median (solid bar) and limit of detection (line) for each experiment is indicated. Data are representative of two independent experiments with 5 mice each. * indicates statistical significance by Mann-Whitney Test (P<0.05).

Defects in both cell wall homeostasis and growth on cytosolic carbon sources suggested that PrkA may be important for intracellular replication and cytosolic survival. To test this hypothesis, we infected bone marrow derived macrophages with wild type *L*. *monocytogenes*, and Δ*prkA* mutants and quantified intracellular growth. Wild type *L*. *monocytogenes* thrived in the macrophage cytosol while the Δ*prkA* mutant not only failed to grow, but by 5 hours post infection began to be killed, displaying a 90% loss in viability between 5 and 8 hours post infection (Fig 2A). Complementation of *prkA* expression *in trans* restored growth in bone-marrow-derived macrophages (BMDMs) (S1C Fig).

The loss of viability from 5–8 hours post infection suggested that Δ*prkA* mutants were being killed in the host cell cytoplasm. To test this hypothesis, we assayed cytosolic bacterial lysis using a luciferase-based bacteriolysis reporter system as previously described [17]. Escape from the phagosome is required for luciferase production as Δ*hly* mutants induced no detectable luciferase production whereas the control strain holin-lysin engineered to lyse upon entry in to the host cytosol lyses ~100-fold more frequently than wild type *L*. *monocytogenes*, respectively (Fig 2B) [17]. Despite significantly decreased bacterial loads, Δ*prkA* mutants lyse ~7-fold more frequently that wild type bacteria in the host cytosol (Fig 2B) consistent with lysis levels of *L*. *monocytogenes* mutants previously described to lyse in the macrophage cytosol [17].

Bacteriolysis of Δ*prkA* mutants in the host cell cytosol suggests that PrkA may be required for avoiding detection by the AIM2 inflammasome and subsequent pyroptotic host cell death [17]. To test this hypothesis, we quantified cell death of macrophages following infection with wild type *L*. *monocytogenes* and Δ*prkA* mutants. As previously described, wild type bacteria induced very limited host cell death while bacteria that lyse in the cytoplasm induced high levels of host cell death (Fig 2C). Infection with Δ*prkA* mutants resulted in significantly increased host cell death; albeit not to the levels induced by the holin-lysin strain (Fig 2C). As expected, complementation of *prkA* expression reduced host cell death to wild-type levels (S1D Fig).

Recent reports suggest that host guanylate-binding proteins (GBPs) play a critical role in cytosolic bacteriolysis of *Francisella tularensis* subsp. *novicida* [13]. To determine if GBPs were required for *L*. *monocytogenes*, and more specifically Δ*prkA* mutant cytosolic bacteriolysis, we infected Gbp^Chr3^ KO macrophages and assayed for host cell death. GBP deficiency did not result in decreases in host cell death following infection with any strains of *L*. *monocyctogenes*. Instead, we observed small, but statistically significant increases in host cell death following infection with strains that activate the AIM2 inflammasome including wild type, holin-lysin and Δ*prkA L*. *monocytogenes*. Importantly, strains that induce either Naip5/Nlrc4 inflammasome activation (L.p.-FlaA) [21] or inflammasome independent cell death (LLO S44A L461T) [55] (Fig 2C) did not demonstrate elevated cell death in GBP deficient macrophages, suggesting that in the context of *L*. *monocytogenes* infection GBPs may act as negative regulators of AIM2 inflammasome activation.

Given its role in intracellular growth, cytosolic survival, inflammasome avoidance, and general cell wall and metabolic stress responses, we hypothesized that PrkA would be required for *L*. *monocytogenes* virulence. To test this hypothesis, mice were challenged with 1x10^5^ *L*. *monocytogenes* wild-type or Δ*prkA* mutants, bacterial burdens in spleens and livers were measured two days post infection. Wild type infected mice harbored high burdens of bacteria in both their livers and their spleens with bacterial burdens reaching 10^8^ bacteria per organ. Strikingly, Δ*prkA* mutants were essentially cleared, demonstrating 4–5 logs of attenuation (Fig 2D). A constitutive expression complementation construct was able to significantly rescue virulence *in vivo* (S1E Fig). Δ*prkA* mutants demonstrated lysozyme sensitivity, suggesting that their virulence defect could be due to killing in the blood during intravenous infection. To test this hypothesis, we assayed killing of wild type, Δ*prkA* mutants in whole blood [51]. While previously described lysozyme sensitive Δ*pgdA*/Δ*oatA* mutants [56] were killed rapidly in whole blood, wild type *L*. *monocytogenes* and Δ*prkA* mutants survived (S3 Fig), suggesting that sensitivity to lysozyme in the blood is not likely responsible for the virulence defects observed *in vivo*. Taken together, these data suggest that PrkA is required for intracellular growth, cytosolic survival avoidance of the AIM2 inflammasome and ultimately virulence *in vivo*.

### Deletion of the putative PrkA substrate YvcK phenocopies Δ*prkA* mutants

**Fig 3:**
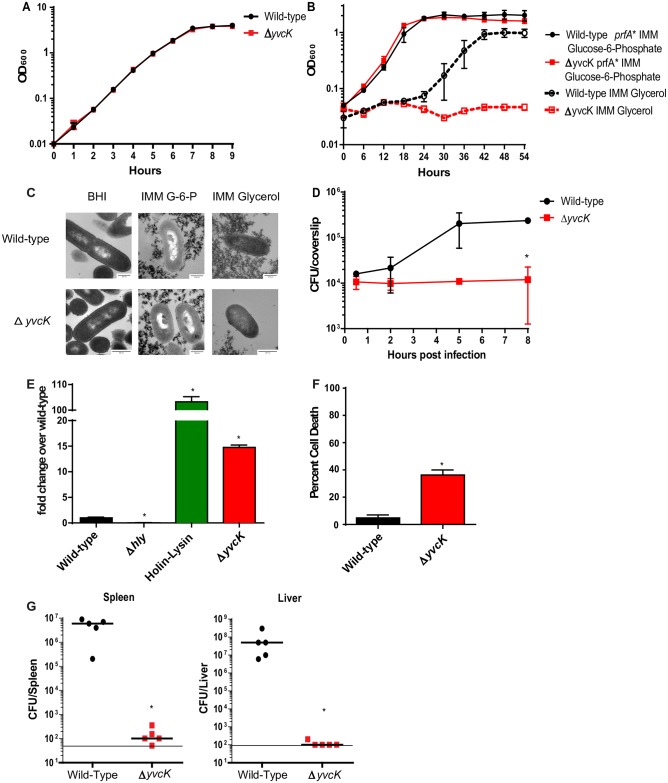
Δ*yvcK* mutants phenocopy Δ*prkA* mutants *in vitro*, *ex vivo* and *in vivo*. **(A**) Growth of the Δ*yvcK* mutant in BHI, **(B)** or in IMM with glucose-6-phosphate (filled symbols and solid lines) or glycerol (open symbols and dashed lines) as the sole carbon source. Data are averages of three biological replicates and error bars represent standard deviation of the mean. **(C)** Morphology of wild-type and the Δ*yvcK* mutant in BHI, IMM Glucose-6-phosphate, and IMM Glycerol at OD 0.5 (BHI) or 6 hours post inoculation. **(D)** Intracellular growth of wild-type (black circles) and Δ*yvcK* mutants (red squares) was determined in bone marrow-derived macrophages (BMDMs) following infection at an MOI of 0.2. **(E)** Intracellular lysis of *L*. *monocytogenes* was measured in immortalized IFNAR^-/-^ macrophages. Macrophages were infected at an MOI of 5 by *L*. *monocytogenes* strains carrying pBHE573 and macrophage luciferase expression was measured 6 hours post infection. **(D, E)** Mean values are reported. Experiments are representative of three or more biological replicates and error bars represent the standard deviation of technical triplicates. * indicates statistical significance by Student’s T-test (P<0.05). **(F)** Host Cell Death induced in wild-type C57/Bl6 BMDMs was analyzed by lactate dehydrogenase release 6 hours post infection at an MOI of 5. Data is an average of three biological replicates and error bars represent standard deviation of the mean. * indicates statistical significance by Student’s T-test (P<0.05). **(G)** C57Bl6 mice were infected intravenously with 1x10^5^ wild-type (black circles) or Δ*yvcK* mutants (red squares) *in vivo*. Spleens (left) and Livers (right) were harvested 48 hours post infection homogenized and plated for CFU. The median (solid bar) and limit of detection (line) for each experiment is indicated. Data are representative of two independent experiments with 5 mice each. * indicates statistical significance by Mann-Whitney Test (P<0.05).

To begin to understand how PrkA regulates virulence potential, we hypothesized that specific PrkA substrates may regulate metabolic or cell wall stress responses required for virulence in vivo. The requirement of PrkA for intracellular growth, cytosolic survival and evasion of the inflammasome was reminiscent of phenotypes previously ascribed to a Δ*yvcK* mutant [17]. Furthermore, recent reports demonstrated that in *M*. *tuberculosis* and *B*. *subtilis*, YvcK homologues are PASTA kinase substrates [36,47]. We hypothesized that YvcK may also be involved in cell wall homeostasis and/or carbon metabolism. Indeed, in *M*. *tuberculosis* and *B*. *subtilis*, YvcK is required for growth on gluconeogenic substrates and for maintenance of cell wall homeostasis. We evaluated the sensitivity of the Δ*yvcK* mutant to the same cell wall stresses as previously described for the Δ*prkA* mutant (Table 1). As was previously described, Δ*yvcK* mutants were hypersusceptible to lysozyme in BHI similar to Δ*prkA* mutants, a phenotype that could be complemented by inducible expression of *yvcK* (S4A Fig) [57]. Additionally, although the magnitude of sensitivity was not as severe as in the Δ*prkA* mutant, Δ*yvcK* mutants were hypersusceptible to all of the same cell wall stresses with the exception of tunicamycin (Table 1).Despite increased sensitivity to some cell wall acting agents, no change in cell wall thickness was observed in the Δ*yvcK* mutant (24.2 ±1.43 nm wild-type vs 25.0 ±0.56 nm Δ*yvcK*) consistent with our previous observations with a Δ*prkA* mutant. Similarly, although Δ*yvcK* mutants grew normally in rich media or minimal media with glucose-6-phosphate as the primary carbon source, they demonstrated severe growth defects in minimal media with glycerol as the primary carbon source (Fig 3A and 3B). The growth defect in glycerol could be complemented by expression of *yvcK in trans* (S4B Fig). In minimal media with glycerol we also observed instances of severe morphology defects in the Δ*yvcK* mutant and other morphology changes similar to the Δ*prkA* mutant (Fig 3C).

Given the similarities in phenotypes between the Δ*prkA* and Δ*yvcK* phenotypes *in vitro*, we assessed virulence of Δ*yvcK* mutants ex vivo and in vivo. Again, consistent with both with the phenotypes observed with a Δ*prkA* mutant and the phenotypes previously reported, Δ*yvcK* mutants were attenuated for intracellular replication (Fig 3D), cytosolic survival (3E) and avoidance of inflammasome activation (3F). Complementation *in trans* restored intracellular growth and host cell death to wild-type levels (S4C and S4D Fig). Finally, Δ*yvcK* mutants were also severely attenuated for virulence in a murine model of disseminated Listeriosis (Fig 3G) and attenuation could be rescued by constitutively expressed *yvcK* (S4E Fig). Taken together these data suggest that YvcK, like PrkA, is required for cell wall homeostasis, glycerol metabolism and virulence both *ex vivo* and *in vivo*.

### YvcK is a substrate of PrkA in *L*. *monocytogenes*

**Fig 4:**
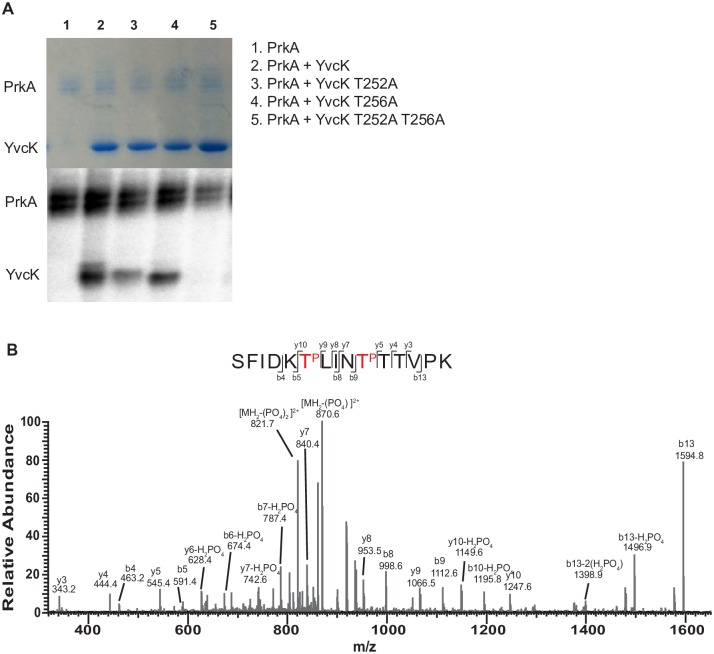
PrkA phosphorylates YvcK on 2 independent threonines. **(A)** PrkA was incubated with [γ-^32^P]ATP separately (lane 1), with YvcK (lane 2) or with YvcK point mutants (lanes 3–5) overnight, proteins were separated by SDS-PAGE and analyzed either by coomasie to demonstrate equivalent amounts of YvcK in each reaction (Top) or autoradiography (Bottom). **(B)** MS/MS of phosphorylated YvcK identified the doubly charged ion 693.34 m/z (monoisotopic mass 1384.67 Da) matched to a doubly phosphorylated YvcK 247–261 tryptic peptide with one missed cleavage site. Phosphorylated threonines (red) 252 and 256 can be identified definitively by either b or y ions.

We next hypothesized, based on previous reports in *B*. *subtilis* and *M*. *tuberculosis*, combined with the congruent observations with the Δ*prkA* and Δ*yvcK* mutants, that YvcK would be a substrate of the PrkA kinase in *L*. *monocytogenes*. To test this hypothesis, we performed *in vitro* phosphorylation assays with [γ-^32^P]ATP using purified His-tagged YvcK and GST-tagged PrkA. *L*. *monocytogenes* YvcK is phosphorylated by PrkA *in vitro*, (Fig 4A) and subsequent MS/MS of the phosphorylated YvcK indicated that, unlike what was previously observed in *M*. *tuberculosis* and *B*. *subtilis*, there were two independent sites of phosphorylation on YvcK, threonine 252 and threonine 256 (Fig 4B). Not only was the double phosphorylation unique, but the phosphorylation sites map to a different location on the predicted tertiary structure of the protein compared to *M*. *tuberculosis* and *B*. *subtilis* whose phosphorylated threonines are located close to the C-terminus. To confirm the sites of phosphorylation, we constructed single or double threonine to alanine phosphoablative point mutants at T252 and T256. Both single point mutants were phosphorylated, although the T252A mutation reduced phosphorylation to a greater extent than the T256A mutation. The double T252A/T256A mutation completely abolished phosphorylation (Fig 4A). Mapping of the PrkA autophosphorylation sites revealed phosphorylation at serine 62, threonine 290, and threonine 308 as sites of autophosphorylation with 91% coverage of the predicted cytosolic region of the protein (S5A–S5C Fig). The PrkA T290 and T308 phosphorylation sites map to a putative unstructured region between the kinase domain and the membrane spanning region that is consistent with previous autophosphorylation sites in *M*. *tuberculosis* [58]. While enriching for phosphopeptides yielded two additional autophosphorylation sites on PrkA S213 and T289 (S2D and S2E Fig), no additional sites were revealed on YvcK. Additionally, we expected to observe phosphorylation in the putative activation loop, as this has been observed for the PASTA kinases in *M*. *tuberculosis* and *Bacillus anthracis* [58,59]. Although we did observe a quadruply phosphorylated fragment ion that contained the putative loop region, the large size of the fragment ion prevented our ability to map specific phosphorylation sites. Taken together these data suggest that YvcK is a PrkA substrate in *L*. *monocytogenes* and indicate novel phospho-regulatory sites on both PrkA and YvcK.

### Regulation of YvcK phosphorylation is dispensable for *in vitro* stress responses but is essential for virulence *ex vivo* and *in vivo*

**Fig 5:**
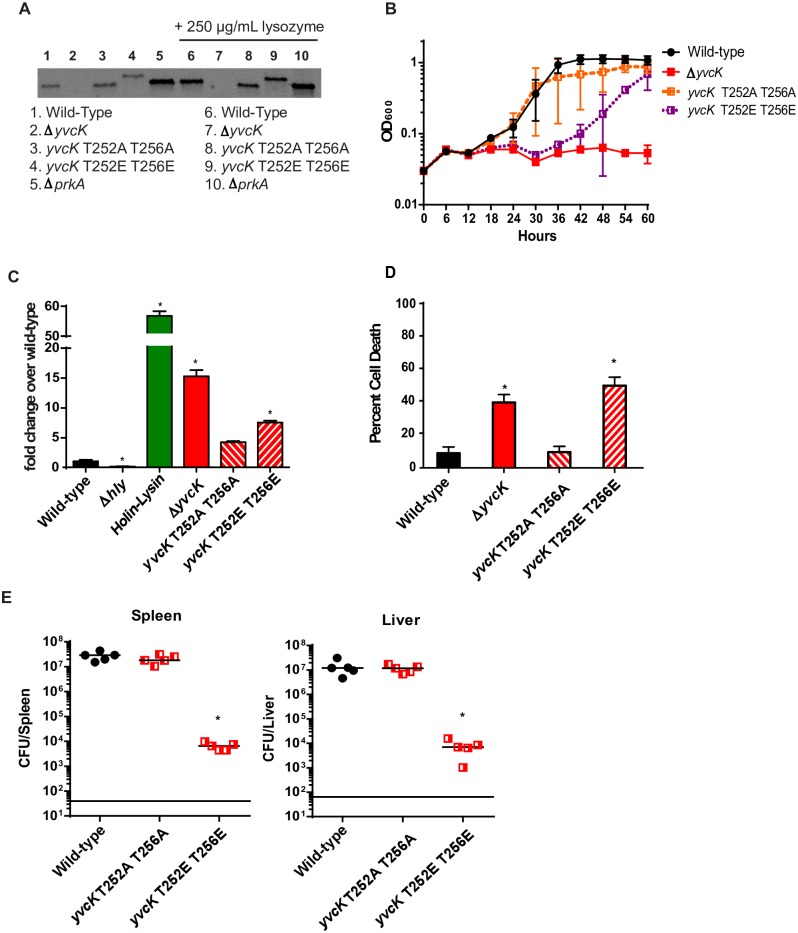
Phosphomimetic YvcK mutants phenocopy Δ*yvcK* mutant *ex vivo* and *in vivo*, but not *in vitro*. **(A**) Expression of YvcK in wild-type, Δ*yvcK*, *yvcK* T252A T256A, *yvcK* T252E T256E, and Δ*prkA* at mid-log in BHI (1–5) or with 250 μg/mL (6–10). **(B)** Growth of wild-type (black circles), *yvcK* T252A T256A (orange squares filled on the left side), *yvcK* T252E T256E (purple squares filled on the right side) in IMM with glycerol as the sole carbon source. Data are averages of three biological replicates and error bars represent standard deviation of the mean. **(C)** Intracellular lysis of *L*. *monocytogenes* was measured in immortalized IFNAR^-/-^ macrophages. Macrophages were infected at an MOI of 5 by *L*. *monocytogenes* strains carrying pBHE573 and macrophage luciferase expression was measured 6 hours post infection. Mean values are reported. Experiments are representative of three or more biological replicates and error bars represent the standard deviation of technical triplicates. * indicates statistical significance by Student’s T-test (P<0.05). **(D)** Host Cell Death induced in wild-type C57/Bl6 BMDMs was analyzed by lactate dehydrogenase release 6 hours post infection at an MOI of 5. Data is an average of three biological replicates and error bars represent standard deviation of the mean. * indicates statistical significance by Student’s T-test (P<0.05). **(E)** C57Bl6 mice were infected intravenously with 1x10^5^ wild-type (black circles), *yvcK* T252A T256A (red squares filled on the left side), *yvcK* T252E T256E (red squares filled on the right side) *in vivo*. Spleens (left) and Livers (right) were harvested 48 hours post infection homogenized and plated for CFU. The median (solid bar) and limit of detection (line) for each experiment is indicated. Data are representative of two independent experiments with 5 mice each. * indicates statistical significance by Mann-Whitney Test (P<0.05).

Given the conserved phenotypes of the Δ*prkA* and Δ*yvcK* mutants, combined with the observation that PrkA phosphorylates YvcK, we hypothesized that phosphorylation of YvcK by PrkA would influence virulence. To test this hypothesis we generated phosphoablative T252A/T256A and phosphomimetic T252E/T256E point mutants in the native *yvcK* locus and assayed glycerol metabolism, cell wall stress responses, cytosolic survival and inflammasome activation *ex vivo* and ultimately virulence *in vivo*. Both the ablative and mimetic mutant versions of YvcK were expressed at similar levels to wild type YvcK as indicated by western blot, although the mimetic version of the protein migrates slightly slower in SDS-PAGE (Fig 5A). We observed upregulation of YvcK in the Δ*prkA* background and when strains were grown in a sub-inhibitory concentration of lysozyme (Fig 5A). We found that mutation of the phosphorylated threonines to either phosphoablative alanines or phosphomimetic glutamic acids had minimal effects on the ability of *L*. *monocytogenes* to withstand cell wall stresses *in vitro* (Table 2). The effects phospho-mutations during metabolic stress were more nuanced as the phosphoablative mutant phenocopied wild type *L*. *monocytogenes* while the phosphomimetic mutants demonstrated a moderate growth defect in glycerol, though not to the level of a full Δ*yvcK* or Δ*prkA* mutant (Fig 5B). Even more surprisingly, we found that the T-A phosphoablative YvcK mutant was indistinguishable from wild type when we assessed cytosolic survival (Fig 5C), inflammasome avoidance (Fig 5D) or virulence *in vivo* (Fig 5E), whereas the T-E phosphomimetic mutant essentially phenocopied a Δ*yvcK* mutant. Taken together, these data suggest that phosphorylation of YvcK inactivates at least some functions of the protein and that regulation of the YvcK phosphorylation state is critical for the virulence of *L*. *monocytogenes*. Furthermore, given that the phosphomimetic mutations did not affect cell wall stress responses, these data suggest that the attenuation of the Δ*yvcK* mutant is unlikely to be due to defects in cell wall stress responses. The condition(s) under which YvcK phosphorylation is beneficial remain to be determined. Similarly, the substrates of PrkA that mediate cytosolic survival and ultimately virulence in vivo are similarly yet to be discovered.

**Table 2:** Minimum inhibitory concentrations of cell wall targeting agents in BHI. Values are mean minimum inhibitory concentrations and standard deviations in μg/mL. Values were determined by three or more biological replicates of serial 2-fold dilutions up or down from 1 μg/mL. Shaded boxes are statistically significant compared to wild-type (Student’s T-Test P< 0.05).

	Wild-Type	*yvcK* T252A T256A	*yvcK* T252E T256E
Ampicillin	0.417 ± **0.144**	0.333 ± **1**	0.208 ± **0.072**
Bacitracin	256 ± **0**	213.3 ± **73.9**	213.3 ± **73.9**
Ceftriaxone	12 ± **6.928**	13.3 ± **4.619**	6.67 ± **2.31**
LL-37	>128 ± **0**	>128 ± **0**	>128 ± **0**
Lysozyme	>4096 ± **0**	>4096 ± **0**	2048 ± **0**

## Discussion

In addition to the classically defined cytosolic pathogens, it is becoming increasingly clear that a number of extracellular and vacuolar pathogens spend at least a portion of their lifecycle in the cytosol of host cells. Furthermore, the identification of extensive arrays of cytosolic innate immune sensing machinery and its role in controlling bacterial infections suggest that the host must survey and defend its cytosol. Indeed, the delivery of non-pathogenic or non-cytosol adapted bacteria leads to their detection and ultimate elimination from the cytosol, however, the mechanisms by which cytosol adapted pathogens sense and respond to the unique environment of the host cytosol is largely unknown. Here, we demonstrate that *L*. *monocytogenes* utilizes its highly conserved PASTA kinase, PrkA, to facilitate cell wall homeostasis, metabolic adaptation, cytosolic survival, inflammasome avoidance and ultimately virulence. Furthermore, we identified a highly conserved protein of unknown function, YvcK, as a substrate of PrkA, which is also required for cell wall homeostasis, metabolic adaptation, cytosolic survival, inflammasome avoidance and ultimately virulence. Finally, although the function of YvcK in metabolic and cell wall stress responses was largely independent of phosphorylation by PrkA, adding phosphomimetic, nonreversible negative charges at the YvcK phosphorylation sites inhibited virulence *ex vivo* and *in vivo*. While the threonine to glutamic acid mutation can sometimes support a relatively simple phosphorylation model, biochemically they are similar but not equivalent [60]. Additionally, the activity of Ser/Thr kinases may be more complicated than an on/off switch [61] which highlights the need to determine substrates under specific environmental conditions. Nevertheless, our data suggest that regulation of YvcK phosphorylation is critical for virulence while additional, yet to be identified substrates of PrkA must be phosphorylated *in vivo* to promote virulence.

A large number of PASTA kinase substrates in a variety of other organism have been identified, however, the complete PrkA phosphoproteome of *L*. *monocytogenes* has not been determined. *Lima et al* previously identified 62 proteins that interact with PrkA, although validation of any of these proteins as bonafide phosphor-substrates of PrkA was not determined [62]. Eight interacting proteins are known PASTA kinase substrates in *B*. *subtilis*, *S*. *pneumonia*, or *M*. *tuberculosis* [35,63–66] while an additional sixteen of these PrkA interacting proteins are also phosphorylated on Ser/Thr residues in *L*. *monocytogenes* [62,67]. Several of these, including PTS system mannose-specific factor IIAB (MptA), fructose-1,6-bisphosphate aldolase (FbaA), glyceraldehyde-3-phosphate dehydrogenase (Gap), pyruvate dehydrogenase (PdhC), and redox-sensing transcriptional repressor (Rex) are directly involved in central metabolism. These data, along with our findings, suggest that PrkA plays a major role in regulating central metabolism in *L*. *monocytogenes*. *Lima et al*. also observed MreB, but not other cytoskeletal proteins such as DivIVA or FtsZ as interacting partners, even though many of these are conserved substrates in multiple other species [36,68,69]. The lack of these proteins in the interactome study may be due to the propensity for these proteins to form insoluble membrane bound complexes during lysis [68,70]. YvcK was also not identified as an interacting protein with PrkA potentially due to a limit of detection issue from low levels of the protein in the extract. Finally, a ActA was previously identified as a PrkA substrate, however the effect of phosphoablative or phosphomimetic mutations has not been determined [62,71]. Although lack of phosphorylation of ActA may explain part of the virulence defect of a Δ*prkA* mutant, Δ*actA* mutants do not have an intracellular growth or lysis defect so phosphorylation of ActA is not likely to be responsible for these phenotypes in a Δ*prkA* mutant [71]. Importantly, as our data demonstrate, identification of phosphorylation events *in vitro*, even when correlated strongly with phenotypes *in vivo*, does not necessarily demonstrate the relevance of phosphorylation *ex vivo* or *in vivo*. As such, we are currently developing novel approaches to characterize the PrkA specific phosphoproteome during intracellular growth.

Regulation of cell wall homeostasis is critical for virulence and PASTA kinases in a variety of other organisms are involved in cell division and cell wall homeostasis [40,72]. Although our results demonstrated that there were no gross defects in cell wall thickness in Δ*prkA* mutants, we cannot rule out the possibility that there are defects specifically in either the abundance of peptidoglycan/wall teichoic acid and/or the structure of these polymers. Indeed, our results suggest that PrkA is selectively involved in multiple, but not all, aspects of cell wall homeostasis as indicated by increased sensitivity of the Δ*prkA* mutant to β-lactams, tunicamycin, antimicrobial peptides and lysozyme but not vancomycin, consistent with the phenotypes of PASTA kinase mutants in *S*. *aureus* and *S*. *pyogenes* [45,73,74]. In *S*. *aureus Δstk1* mutants have reduced levels of UDP-MurNAc as well as other downstream peptidoglycan precursors [75]. β-lactams function by inactivating PBPs and by reducing the peptide crosslinking in peptidoglycan [76,77]. Δ*prkA* mutant sensitivity to β-lactams may be due to changes in peptidoglycan precursors caused by phosphorylation of GlmU [35] or changes in transcription of proteins in the MurA-G operon [78]. Inhibition of MurE and MurF or other early steps in peptidoglycan synthesis cause increased sensitivity to β-lactam antibiotics in *S*. *aureus* [79–81]. Alternatively, the increased β-lactam sensitivity of the Δ*prkA* mutant may be due to mislocalization of PBPs, potentially through the misregulation of MreB, a putative PrkA interacting protein that has been found to be phosphorylated on a serine [62,67,82]. MreB activity has been linked to β-lactam sensitivity [83]. The increased tunicamycin sensitivity of the Δ*prkA* mutant may be caused by perturbations in the peptidoglycan synthesis pathway through its inhibition of MraY [84] or in wall teichoic acid biosynthesis through its inhibition of TagO [84]. Although MraY has not been identified as a PrkA substrate the next enzyme in the pathway (MurG) is a putative interacting partner with PrkA [62] and proteins involved in MraY regulation, DivIVA/Wag31 [85], are conserved PASTA kinase targets. While inhibition of wall teichoic acid (WTA) synthesis through TagO is not detrimental to growth in *S*. *aureus* [86], this may require an unknown compensatory mechanism which is non-functional in a Δ*prkA* mutant. Additionally, in *L*. *monocytogenes*, resistance to antimicrobial peptides is linked to rhamnosylation of WTA [87]. Lmo1081 (RmlA), required for rhamnosylation of WTA, is a PrkA interacting protein and Ser/Thr phosphoprotein [62,67,87], potentially linking the increased sensitivity of the Δ*prkA* mutant to tunicamycin and LL-37. Finally, effect of the Δ*prkA* mutation of lysozyme resistance may be caused by several different mechanisms including peptidoglycan precursor abundance, mislocalization of pbps, or regulation of PgdA and OatA, peptidoglycan modification enzymes [5,56]. Although a detailed understanding of PrkA regulation of cell wall remains to be determined, pharmacologic targeting of PrkA could result in synergistic activity with already existing antimicrobials to provide a new approach to combating gram-positive pathogens [46].

PknB in *M*. *tuberculosis* is essential while Stk1 in *S*. *aureus* is important for growth in some nutrient limiting conditions [88,89]. Consistent with these observations, we found that PrkA is required for growth in minimal media with glycerol as the primary carbon source. This effect may be due to direct modulation of glycolysis/gluconeogenesis by PrkA. Several conserved PASTA kinase substrates identified as both PrkA interactors and Ser/Thr phosphorylated proteins in *L*. *monocytogenes* are glycolytic enzymes [37,62,67]. Phosphorylation of glycolytic enzymes, such as FbaA, Gap, or PykA, could control their activity in order to regulate flux between competing pathways (eg. The pentose phosphate pathway) or to regulate flux between opposing pathways (eg. Gluconeogenesis). Furthermore the master virulence regulator, PrfA, is regulated by [91] and regulates [11,90] central metabolism. How posttranslational regulation of central metabolism by PrkA may affect PrfA activity is unknown. For example, glycerol metabolism is required for optimal PrfA activity, therefore regulation of glycerol metabolism by PrkA may directly affect virulence factor expression. In addition to energy and carbon input into the cell PrkA may also have a major role in regulating energy expenditures. EF-Tu and EF-G are conserved PASTA kinase substrates [66,90] and interacting-Ser/Thr phosphorylated proteins [62,67,91] required for protein translation [92], a process that can account for >50% of the ATP consumption by a cell [93]. Thus PrkA is likely to be crucial for matching energy intake and expenditure of the cell. Intracellular pathogens have evolved specific metabolic strategies to avoid disruption of host glycolysis to avoid innate immune detection and maintain their replicative niche [94]. Therefore, the regulation of central metabolism by PrkA could be considered an essential virulence factor. PrkA may also be important for regulating the production of specific metabolites. PASTA kinase mutants are defective in purine biosynthesis in multiple organisms [75,89,95]. This specific auxotrophy is not likely to be responsible for the PrkA growth defect as adenine is a normal component of IMM but may play a role in growth *in vivo* [8]. Metabolism is essential for bacterial pathogenesis and the use of novel metabolomics approaches will further elucidate how PrkA regulates metabolism to promote virulence.

In both *M*. *tuberculosis* and *B*. *subtilis* YvcK homologues are required for cell wall homeostasis, cell morphology and gluconeogenic metabolism, but the enzymatic function of YvcK remains unknown [47–50]. We found that *L*. *monocytogenes* YvcK was similarly required for growth on glycerol, a gluconeogenic substrate for *L*. *monocytogenes* as well as for cell wall homeostasis and normal morphology. YvcK is similar to the 2-phospho-L-lactate transferase CofD found in *M*. *tuberculosis* and some *Archea* [48,50]. However many of the YvcK containing bacteria do not produce coenzyme F420, the product of CofD [48,50]. Furthermore although structures of CofD and multiple YvcK homologues have been solved, thus far structural analysis has not predicted a specific interacting metabolite for YvcK [96]. In three different bacterial species YvcK has been shown to be important in metabolism and more specifically gluconeogenesis. Although carbon sources utilized for gluconeogenesis by each of these species are diverse, the conserved requirement for YvcK suggests that it regulates a central process in gluconeogenesis. YvcK is a conserved PASTA kinase substrate in several organisms, however, the exact sites and numbers of phosphorylations are different [36,47]. In *M*. *tuberculosis* CuvA (YvcK)-dependent phenotypes were phosphorylation independent [50] whereas in *B*. *subtilis*, cell wall phenotypes were rescued by phosphomimetic mutants [47]. Contrary to both of these results, in *L*. *monocytogenes* phosphoablative YvcK mutants phenocopied wild-type under all conditions whereas phosphomimetic mutations were detrimental for growth in glycerol and virulence *in vivo*. Taken together, our results suggest that while PASTA kinases regulate YvcK homologues in a variety of organisms, the mechanism of regulation is species specific. Additionally, while PASTA kinases are unique to gram-positive organisms, YvcK homologues are found across eubacteria and archaea [48], therefore uncovering YvcK’s metabolic role could have broad implications for our understanding of central metabolism and virulence in a wide variety of YvcK containing pathogens.

We found that both PrkA and its substrate YvcK were required for cytosolic survival and avoidance of the AIM2 inflammasome. The specific stresses leading to bacteriolysis of bacteria in the cytosol of host cells are currently unknown. Recently, Broz and colleagues demonstrated that GBPs were involved in cytosolic lysis of *F*. *novicida* and contributed to AIM2 activation, potentially through direct lysis of cytosolic bacteria [13]. In addition, previous work from Coers and colleagues suggested that GBPs were involved in activation of caspase-11-dependent inflammasome activation [97]. Our analysis of macrophages lacking the GBPs on chromosome 3, including GBP1,2,3,5, and 7 [98], suggests that these GBPs are not required for cytosolic lysis and subsequent inflammasome activation following infection with either wild type *L*. *monocytogenes* or mutants with increased bacteriolysis in the cytosol, including Δ*yvcK* and Δ*prkA* mutants. Counterintuitively, we found that GBP deficient macrophages demonstrated increased host cell death following infection with strains that specifically activated AIM2, suggesting that during *L*. *monocytogenes* infection GBPs act as a negative regulator of AIM2 activation. Given that this effect was only seen with *L*. *monocytogenes* strains that activate the AIM2 inflammasome and not with strains that activate the Naip5/Nlrc4 inflammasome, perhaps GBPs negatively regulate AIM2 inflammsome activation through masking or sequestering of DNA following bacteriolysis. It is possible that the GBPs located on chromosome 5, including GBP4,6,8,9,10 and 11 [98], could be required for *L*. *monocytogenes* bacteriolysis or that *L*. *monocytogenes* lyse due to some other stress or antimicrobial mechanism. Previously, a cationic antimicrobial peptide, ubiquicidin, had been purified from the cytosol of IFNγ activated macrophages and had been demonstrated to have anti-*Listeria* activity in vitro [12]. Similarly, lysozyme has recently been demonstrated to be able to access the cytosol and kill bacteria in this compartment, leading to inflammasome activation [56].Consistent with these as potential causes of cytosolic bacteriolysis, both the Δ*prkA* mutant and the Δ*yvcK* mutant demonstrated increases in LL-37 and lysozyme sensitivity *in vitro*. Finally, Δ*yvcK* mutants and Δ*prkA* mutants demonstrate defects in morphology during growth on cytosolically available carbon sources, suggesting that lysis of these mutants may be due to metabolic defects that result in impaired cell wall homeostasis. Identification of additional PrkA substrates and the specific enzymatic activity of YvcK may further elucidate cell wall homeostasis and/or metabolic pathways required for cytosolic survival and virulence.

In conclusion, we have demonstrated that the *L*. *monocytogenes* PASTA kinase PrkA and its conserved substrate YvcK play essential roles in cell wall homeostasis, metabolism, and ultimately virulence. Surprisingly, despite the exquisite conservation of phenotypes of the two null mutants, phosphorylation appears to inhibit the function of YvcK such that phosphomimetic YvcK mutants are highly attenuated in vivo while phosphoablative mutants phenocopy wild type *L*. *monocytogenes*. Importantly, in addition to identifying two novel essential virulence factors in *L*. *monocytogenes*, our work highlights the importance of identifying PrkA substrates during infection. Finally, given the high conservation of these proteins in a number of important pathogens and their conserved roles in virulence, targeting PASTA kinases and/or YvcK function represents a novel and exciting avenue for the development of new antimicrobials.

## Methods

### Bacterial strains and growth conditions

All *L*. *monocytogenes* strains used were 10403s background and the Δ*yvcK* and Δ*prkA* mutants were previously described [17,46]. The *yvcK* complementation vector pPL2e_riboE_*yvcK* was constructed as previously described [46]. Briefly, the theophylline inducible riboswitch E was added to *yvcK* by SOE PCR [99], and cloned in pPL2e [100]. Point mutations in *yvcK* were made by designing the desired mutations into gBlocks (IDT) and subcloned in pPL2e_riboE_*yvcK*. YvcK inserts from the wild type or mutated pPL2e_riboE_*yvcK* constructs were then used as the source for subsequent cloning into pET20b to facilitate His-tagged purification from Rosetta pLysS *E*.*coli* and into pKSV7 for subsequent reintegration into the native locus of the Δ*yvcK* mutant. Constitutive expression of *yvcK* and *prkA* from the pHelp promoter was achieved by cloning into pIMK2 [101]. For Δ*prkA* complementation, the resulting pHelp_*prkA* construct was cloned into a new pPL1 vector (pPL1k). pPL1k was constructed by removing the chloramphenicol resistance cassette from pPL1 [100] with restriction enzymes ApaLI and PvuI and inserting the kanamycin resistance cassette from pIMK2 [101]. The pPL1k_*prkA* construct was conjugated into a new phage-cured Δ*prkA* strain. For a complete list of strains see S1 Table.

For all assays overnight cultures of *L*. *monocytogenes* strains were grown to stationary phase at 30°C with no shaking in Brain-Heart Infusion (BHI).

### Minimum inhibitory concentration assays

Overnight cultures grown to stationary phase at 30°C with no shaking in BHI media were inoculated at a 1:50 ratio into 96-well plates containing BHI with growth inhibitors in 2 fold serial dilutions up or down from 1μg/mL. Plates were grown at 37°C with continuous shaking for 12 hours in an Eon or Synergy HT Microplate Spectrophotometer (BioTek Instruments, Inc., Winooski, VT) and OD_600_ was read every 15 minutes. The MIC was defined as the lowest concentration at 12 hours that gave an equivalent OD_600_ to the starting inoculum. All growth assays were performed with at least 5 biological replicates and the mean MIC was selected [50].

### Growth assays

Overnight cultures grown to stationary phase at 30°C with no shaking in BHI were washed in PBS and inoculated at a 1:50 ratio into BHI or Improved Minimal Media (IMM) [102] and grown at 37°C with continuous shaking. For BHI OD_600_ was read at 1 hour time points for 9 hours. IMM was made with either 55mM, Glycerol, or Glucose-6-Phosphate. For IMM OD_600_ was read at 6 hour time points for at least 54 hours. All growth assays were performed with 3 biological replicates.

### Microscopy

Cultures were harvested at an OD of 0.5 in BHI or after 6 hours of growth in IMM. Cells were fixed, washed, dried, infiltrated, and sectioned as previously described [103]. Sections were imaged with a Phillips CM120 STEM microscope. Cell wall thickness was measured with ImageJ software. Ten measurements at the mid-cell of ten bacteria were taken for each of 3 biological replicates.

### Intracellular growth curve

Bone marrow derived macrophages (BMDMs) were prepared from C57BL/6 mice as previously described [104]. BMDMs were plated at 5*10^6^ cells per 60mm dish with coverslips and allowed to adhere overnight. BMDMs were infected at an MOI of 0.2 and infection was quantified as previously described [17]. The growth curve is representative of 3 biological replicates.

### Intracellular lysis assay

Intracellular lysis was measured as previously described [17]. Briefly, immortalized INFAR^-/-^ BMDMs [105] were plated at 5*10^5^ cells/well in 24 well plates overnight. Cells were infected at an MOI of ten with strains containing the pBHE573 reporter construct. At 1 hour post infection, media was removed from the plate and replaced with fresh media containing gentamycin. At six hours post infection cells were lysed in TNT lysis buffer. Cell supernatants were mixed with luciferase reagent as previously described. Luciferase activity was measured in a Synergy HT Microplate Spectrophotometer (BioTek Instruments, Inc., Winooski, VT). A representative experiment from 3 biological replicates is shown.

### LDH assay

Induction of host cell death by *L*. *monocytogenes* infection was measured by the lactate dehydrogenase (LDH) assay as previously described [106]. Briefly, 5*10^5^ BMDMs were pre-treated with Pam3CSK4 (Invivogen tlrl-pms) in 24-well plates overnight. BMDMs were infected at a MOI of five. At ½ hour post infection, media was removed from the plate and replaced with fresh media containing gentamycin. For the experiment with LLO S44A L461T gentamycin was removed after an hour. Six hours post infection, macrophage cell death was determined by measuring LDH release into the culture supernatant. 100% lysis was determined by addition of Triton X-100 to a final concentration of 1%. All LDH assays are the average of 3 biological replicates.

### Murine IV infection

Acute mouse IV infections were performed according to IAUCC approved protocol as previously described [21]. Briefly, 6 to 8-week-old female C57BL/6 mice were infected IV with 1×10^5^ CFU. 48 hours post-infection, livers and spleens were harvested, homogenized in PBS with 0.1% NP-40, and plated for CFU. Two independent replicates of each experiment with 5 mice per group were performed.

### Protein production

PrkA was purified as previously described [46]. For purification of YvcK, overnight cultures of Rosetta pLysS pET20b constructs were inoculated at a 1:50 ratio and grown to an OD of ~0.5 at 37°C 250rpm. IPTG was added to a final concentration of 1mM and rpm was lowered to 180. At 3 hours post induction cultures were pelleted, resuspended in PBS, and stored at -80°C. Pellets were thawed, lysed, and pelleted. The supernatant was collected and mixed with NTA-Nickel resin (Pierce) for 30 minutes at 4°C. Resin was pelleted, washed, and the protein was eluted with 250mM Imidazole in 20mM Tris pH 7.4 100mM NaCl. Elutions were concentrated and further purified using a Sephadex 75 size exclusion column (GE Healthcare) on an ÄKTA purifier FPLC (GE Healthcare). Protein was eluted using an isocratic method in a buffer containing 150mM NaCl and 10mM Tris pH 8.0. Non-aggregated fractions indicated by UV absorbance were visualized on SDS-PAGE and fractions of >98% purity were pooled and used for biochemical assays. Protein concentration was determined by BCA assay (Pierce) according to manufacturer’s protocols.

### Phosphorylation assay

Phosphorylation assays were performed by mixing 3 μg of kinase with 2μg YvcK in a 30-μl reaction mixture containing 50 mM Tris-HCl (pH 7.4), 1 mM dithiothreitol (DTT), 5 mM MgCl_2_, 250 μM ATP, and 1 μCi of [γ-^32^P]ATP, followed by incubation at room temperature overnight. The reactions were terminated by the addition of 5× SDS loading buffer to the mixture. Samples were separated by SDS-PAGE, fixed, dried, and analyzed by autoradiography

### Mass spectrometry

Phosphorylation of YvcK was performed with 10μg of kinase and 100μg of YvcK in 100mM ATP in containing 50 mM Tris-HCl (pH 7.4), 1 mM dithiothreitol (DTT), 5 mM MnCl_2_. The reaction was digested with trypsin. Digests were cleaned with OMIX C18 SPE cartridges (Agilent, Palo Alto, CA) according to the manufacturer’s protocol. Where indicated, phosphopeptides were enriched with titanium dioxide coated beads and eluted from the beads with ammonium hydroxide. Peptides were analyzed by nanoLC-MS/MS with a Agilent 1100 nanoflow system coupled to a hybrid linear ion trap-orbitrap mass spectrometer (LTQ-Orbitrap Elite, Thermo Fisher Scientific) equipped with an EASY-Spray electrospray source. Raw MS/MS data was converted to mgf file format and used to search against the *L*. *monocytogenes* RefSeq database with a list of common lab contaminants using the *Mascot* search engine 2.2.07 (Matrix Science). Protein annotations, significance of identification, and spectral based quantification was done with help of Scaffold software (version 4.3.2, Proteome Software Inc., Portland, OR).

### PrkA inhibition assay

Overnight cultures grown to stationary phase at 30°C with no shaking in BHI media were inoculated at a 1:50 ratio into 96-well plates containing BHI with Staurosporine at 20μM. Plates were grown at 37°C with continuous shaking for 12 hours in an Eon or Synergy HT Microplate Spectrophotometer (BioTek Instruments, Inc., Winooski, VT) and OD6_00_ was read every hour. Growth curve is representative of 3 biological replicates.

### Western blot

Overnight cultures grown to stationary phase at 30°C with no shaking in BHI were inoculated at a 1:50 ratio into BHI with or without 250μg lysozyme and grown to an OD of 0.5 at 37°C shaking. 10mLs of culture was pelleted, washed in PBS, and resuspended in lysis buffer (50mM Tris pH 7.4, 5mM DTT, 0.1% SDS). Pellets were bead beat for 2 minutes and beads and cell debris were pelleted. Lysate was filtered through a 0.2 micron filter and total protein level was quantified by BCA assay. Equivalent protein concentrations were run on a SDS-PAGE gel and transferred to a Hybond-ECL membrane (GE). Custom polyclonal anti-YvcK antibody was used to assess protein levels together with 2° anti-rabbit DyLight 800 and a Li-Cor Odyssey 9120. Quantification was performed with attached Odyssey software.

### Ethics statement

Mice were cared for according to the recommendations of the NIH, published in the Guide for the Care and Use of Laboratory Animals. All techniques used were reviewed and approved by the University of Wisconsin-Madison Institutional Animal Care and Use Committee (IACUC) under the protocol M02501.

### Statistical analysis

Prism 6 (GraphPad Software) was used for statistical analysis of data. Means from two groups were compared with unpaired two-tailed Student’s T-test. Means from more than two groups were analyzed by one-way ANOVA with a post-hoc LSD Test. Medians from two groups were compared with Mann-Whitney Test. * indicates a statistically significant difference (P is less than 0.05).

## Supporting Information

S1 Fig Cell wall and metabolic defects of the Δ*prkA* mutant can be complemented *in trans*. **(A)** Growth of wild-type (black circles) and the Δ*prkA* pPL2e_riboE_*prkA* mutant (blue triangles) in 0.0625μg/mL tunicamycin with or without 2mM Theophylline for complementation. Overnight cultures in BHI were grown in the absence (filled symbols and solid lines) or presence (open symbols and dashed lines) of 2mM Theophylline and back-diluted 1:50 into 96-well plates containing the same Theophylline concentration. Plates were grown at 37°C with continuous shaking for 12 hours in an Eon or Synergy HT Microplate Spectrophotometer (BioTek Instruments, Inc., Winooski, VT) and OD_600_ was read every hour. Growth curves are representative of 3 biological replicates. **(B)** Growth of wild-type (black circles) and the Δ*prkA* pPL2e_riboE_*prkA* mutant (blue triangles) in IMM Glycerol without (filled symbols and solid lines) or with 2mM Theophylline (open symbols and dashed lines) for complementation. Overnight BHI cultures were washed, inoculated into minimal media, grown at 37°C, and OD_600_ was measured every 6 hours. **(C)** Intracellular growth of wild-type (black circles) and Δ*prkA* mutants (blue triangles) was determined in bone marrow-derived macrophages (BMDMs) in media containing 1mM theophylline following infection at an MOI of 0.2. **(D)** Host cell death induced by wild-type (black) and Δ*prkA* pPL2e_riboE_*prkA* (blue) in uninduced (solid) or theophylline induced wells (checkered). BMDMs were infected with an MOI of 5 and complementation wells were maintained in 1mM Theophylline. Media supernatant was harvested at 6 hours and assayed for lactate dehydrogenase (LDH) activity from lysed macrophages. **(E)** C57Bl6 mice were infected intravenously with 1x10^5^ wild-type (black circles), Δ*prkA* mutants (blue triangles), or Δ*prkA* pPL1k *prkA* (empty blue triangles) *in vivo*. Spleens (left) and Livers (right) were harvested 48 hours post infection homogenized and plated for CFU. The median (solid bar) and limit of detection (line) for each experiment is indicated. Data are representative of two independent experiments with 5 mice each. * indicates statistical significance by Mann-Whitney Test (P<0.05).
(TIF)

S2 Fig Δ*prkA* and Δ*yvcK* mutants display morphology defects in minimal media with glycerol. Morphology of wild-type, Δ*prkA* mutant, and Δ*yvcK* mutant in BHI, IMM Glucose-6-phosphate, and IMM Glycerol at OD 0.5 (BHI) or 6 hours post inoculation.
(TIF)

S3 Fig The Δ*prkA* and Δ*yvcK* mutants have no survival defect in blood. Survival of wild-type (black circles), Δ*pgdA ΔoatA* (purple diamonds), Δ*yvcK* (red squares), and Δ*prkA* (blue triangles) in defribronated sheep’s blood. Blood was inoculated with 5*10^6^ CFU, incubated at 37°C, and plated for CFU at specified time points. Values are an average of 3 biological replicates and error bars represent standard deviation of the mean.
(TIF)

S4 Fig Cell wall and metabolic defects of the Δ*yvcK* mutant can be complemented *in trans*. **(A**) Growth of wild-type (black circles) and the Δ*yvcK* pPL2e_riboE_*yvcK* mutant (red squares) in 1024μg/mL Lysozyme with or without 2mM Theophylline for complementation. Overnight cultures in BHI were grown in the absence (filled symbols and solid lines) or presence (open symbols and dashed lines) of 2mM Theophylline and back-diluted 1:50 into 96-well plates containing the same Theophylline concentration. Plates were grown at 37°C with continuous shaking for 12 hours in an Eon or Synergy HT Microplate Spectrophotometer (BioTek Instruments, Inc., Winooski, VT) and OD_600_ was read every hour. Growth curves are representative of 3 biological replicates. **(B)** Growth of wild-type (black circles) and the Δ*yvcK* pPL2e_riboE_*yvcK* mutant (red squares) in IMM Glycerol without (filled symbols and solid lines) or with 2mM Theophylline (open symbols and dashed lines) for complementation. Overnight BHI cultures were washed, inoculated into minimal media, grown at 37°C, and OD_600_ was measured every 6 hours. **(C)** Intracellular growth of wild-type (black circles) and Δ*yvcK* mutants (red squares) was determined in bone marrow-derived macrophages (BMDMs) in media containing 1mM theophylline following infection at an MOI of 0.2. **(D)** Host cell death induced by wild-type (black) and Δ*yvcK* pPL2e_riboE_*yvcK* (red) in uninduced (solid) or theophylline induced wells (checkered). BMDMs were infected with an MOI of 5 and complementation wells were maintained in 1mM Theophylline. Media supernatant was harvested at 6 hours and assayed for lactate dehydrogenase (LDH) activity from lysed macrophages. **(E)** C57Bl6 mice were infected intravenously with 1x10^5^ wild-type (black circles), Δ*yvcK* pIMK2 empty (red squares), or Δ*yvcK* pIMK2 *yvcK* (empty red squares) *in vivo*. Spleens (left) and Livers (right) were harvested 48 hours post infection homogenized and plated for CFU. The median (solid bar) and limit of detection (line) for each experiment is indicated. Data are representative of two independent experiments with 5 mice each. * indicates statistical significance by Mann-Whitney Test (P<0.05).
(TIF)

S5 Fig PrkA autophosphorylates itself on multiple sites. MS/MS spectra of PrkA phosphopeptides. S64 **(A)**, T289 **(B)**, and T307 **(C)** were identified as autophosphorylation sites from three separate tryptic peptides. S64 phosphorylation in peptide 58–96 can be identified definitively solely through b ions. T289 phosphorylation in peptide 280–298 can be identified definitively through the combination of b and y ions. T307 phosphorylation in peptide 299–313 can be identified definitively by either b or y ions. Phosphopeptides identified post titanium oxide enrichment identified S213 **(D)** and T268 **(E)** as autophosphorylation sites. S213 phosphorylation and T268 phosphorylation in peptides 207–220 and 265–272 can be identified definitively by either b or y ions.
(TIF)

S1 Table Strains used in this study. *L*. *monocytogenes* (top) and *E*. *coli* (bottom) strains used in this study. Strain designation, genotype, and source is listed left to right.
(XLSX)

## References

[1] Bayles KW, Wesson CA, Liou LE, Fox LK, Bohach GA, Trumble WR. Intracellular *Staphylococcus aureus* Escapes the Endosome and Induces Apoptosis in Epithelial Cells. Infect Immun. 1998;66: 336–342. 9423876 10.1128/iai.66.1.336-342.1998 PMC107895

[2] van der Wel N, Hava D, Houben D, Fluitsma D, van Zon M, Pierson J, et al *M*. *tuberculosis* and *M*. *leprae* translocate from the phagolysosome to the cytosol in myeloid cells. Cell. 2007;129: 1287–98. 10.1016/j.cell.2007.05.059 17604718

[3] Portnoy DA, Jacks PS, Hinrichs DJ. Role of Hemolysin for the Intracellular Growth OF *Listeria monocytogenes* . J Exp Med. 1988;167: 1459–1471. 2833557 10.1084/jem.167.4.1459 PMC2188911

[4] Santic M, Molmeret M, Klose KE, Jones S, Abu Kwaik Y. The *Francisella tularensis* pathogenicity island protein IgIC and its regulator MgIA are essential for modulating phagosome biogenesis and subsequent bacterial escape into the cytoplasm. Cell Microbiol. 2005;7: 969–979. 10.1111/j.1462-5822.2005.00526.x 15953029

[5] Aubry C, Goulard C, Nahori M-A, Cayet N, Decalf J, Sachse M, et al OatA, a peptidoglycan O-acetyltransferase involved in *Listeria monocytogenes* immune escape, is critical for virulence. J Infect Dis. 2011;204: 731–40. 10.1093/infdis/jir396 21844299 PMC3156107

[6] Peng K, Broz P, Jones J, Joubert LM, Monack D. Elevated AIM2-mediated pyroptosis triggered by hypercytotoxic *Francisella* mutant strains is attributed to increased intracellular bacteriolysis. Cell Microbiol. 2011;13: 1586–1600. 10.1111/j.1462-5822.2011.01643.x 21883803 PMC3173570

[7] O’Riordan M, Moors MA, Portnoy DA. *Listeria* intracellular growth and virulence require host-derived lipoic acid. Science. 2003;302: 462–464. 10.1126/science.1088170 14564012

[8] Pechous R, Celli J, Penoske R, Hayes SF, Frank DW, Zahrt TC. Construction and characterization of an attenuated purine auxotroph in a *Francisella tularensis* live vaccine strain. Infect Immun. 2006;74: 4452–4461. 10.1128/IAI.00666-06 16861631 PMC1539594

[9] Goetz M, Bubert A, Wang G, Chico-Calero I, Vazquez-Boland JA, Beck M, et al Microinjection and growth of bacteria in the cytosol of mammalian host cells. Proc Natl Acad Sci U S A. 2001;98: 12221–6. 10.1073/pnas.211106398 11572936 PMC59795

[10] Brumell JH, Rosenberger CM, Gotto GT, Marcus SL, Finlay BB. SifA permits survival and replication of *Salmonella typhimurium* in murine macrophages. Cell Microbiol. 2001;3: 75–84. 10.1046/j.1462-5822.2001.00087.x 11207622

[11] Chico-Calero I, Suárez M, González-Zorn B, Scortti M, Slaghuis J, Goebel W, et al Hpt, a bacterial homolog of the microsomal glucose- 6-phosphate translocase, mediates rapid intracellular proliferation in *Listeria* . Proc Natl Acad Sci U S A. 2002;99: 431–436. 10.1073/pnas.012363899 11756655 PMC117577

[12] Hiemstra PS, van den Barselaar MT, Roest M, Nibbering PH, van Furth R. Ubiquicidin, a novel murine microbicidal protein present in the cytosolic fraction of macrophages. J Leukoc Biol. 1999;66: 423–428. 10496312 10.1002/jlb.66.3.423

[13] Meunier E, Wallet P, Dreier RF, Costanzo S, Anton L, Rühl S, et al Guanylate-binding proteins promote activation of the AIM2 inflammasome during infection with *Francisella novicida* . Nat Immunol. 2015;16: 476–486. 10.1038/ni.3119 25774716 PMC4568307

[14] Man SM, Karki R, Malireddi RKS, Neale G, Vogel P, Yamamoto M, et al The transcription factor IRF1 and guanylate-binding proteins target activation of the AIM2 inflammasome by *Francisella* infection. Nat Immunol. 2015;16: 467–75. 10.1038/ni.3118 25774715 PMC4406811

[15] Witte CE, Archer KA, Rae CS, Sauer J-D, Woodward JJ, Portnoy DA. Innate immune pathways triggered by *Listeria monocytogenes* and their role in the induction of cell-mediated immunity. Advances in immunology. 1st ed. Elsevier Inc.; 2012 pp. 135–56. 10.1016/B978-0-12-394590-7.00002-6 22244582

[16] Finlay BB, McFadden G. Anti-immunology: Evasion of the host immune system by bacterial and viral pathogens. Cell. 2006;124: 767–782. 10.1016/j.cell.2006.01.034 16497587

[17] Sauer J-D, Witte CE, Zemansky J, Hanson B, Lauer P, Portnoy DA. *Listeria monocytogenes* triggers AIM2-mediated pyroptosis upon infrequent bacteriolysis in the macrophage cytosol. Cell Host Microbe. Elsevier Ltd; 2010;7: 412–9. 10.1016/j.chom.2010.04.004 20417169 PMC2947455

[18] Hansen K, Prabakaran T, Laustsen A, Jørgensen SE, Rahbæk SH, Jensen SB, et al *Listeria monocytogenes* induces IFNβ expression through an IFI16-, cGAS- and STING-dependent pathway. EMBO J. 2014;33: 1654–66. 10.15252/embj.201488029 24970844 PMC4194099

[19] Hornung V, Ablasser A, Charrel-Dennis M, Bauernfeind F, Horvath G, Caffrey DR, et al AIM2 recognizes cytosolic dsDNA and forms a caspase-1-activating inflammasome with ASC. Nature. Nature Publishing Group; 2009;458: 514–518. 10.1038/nature07725 19158675 PMC2726264

[20] Miao EA, Leaf IA, Treuting PM, Mao DP, Dors M, Sarkar A, et al Caspase-1-induced pyroptosis is an innate immune effector mechanism against intracellular bacteria. Nat Immunol. Nature Publishing Group; 2010;11: 1136–1142. 10.1038/ni.1960 21057511 PMC3058225

[21] Sauer J-D, Pereyre S, Archer KA, Burke TP, Hanson B, Lauer P, et al *Listeria monocytogenes* engineered to activate the Nlrc4 inflammasome are severely attenuated and are poor inducers of protective immunity. Proc Natl Acad Sci U S A. 2011;108: 12419–24. 10.1073/pnas.1019041108 21746921 PMC3145703

[22] Jorgensen I, Miao EA. Pyroptotic cell death defends against intracellular pathogens. Immunol Rev. 2015;265: 130–142. 10.1111/imr.12287 25879289 PMC4400865

[23] Swaminathan B, Gerner-Smidt P. The epidemiology of human listeriosis. Microbes Infect. 2007;9: 1236–1243. 10.1016/j.micinf.2007.05.011 17720602

[24] McCollum JT, Cronquist AB, Silk BJ, Jackson KA, O’Connor KA, Cosgrove S, et al Multistate outbreak of listeriosis associated with cantaloupe. N Engl J Med. 2013;369: 944–53. 10.1056/NEJMoa1215837 24004121

[25] Hamon M, Bierne H, Cossart P. *Listeria monocytogenes*: a multifaceted model. Nat Rev Microbiol. 2006;4: 423–434. 10.1038/nrmicro1413 16710323

[26] Cossart P, Toledo-Arana A. *Listeria monocytogenes*, a unique model in infection biology: an overview. Microbes Infect. Elsevier Masson SAS; 2008;10: 1041–1050. 10.1016/j.micinf.2008.07.043 18775788

[27] Gaillard JL, Berche P, Frehel C, Gouin E, Cossart P. Entry of *L*. *monocytogenes* into cells is mediated by internalin, a repeat protein reminiscent of surface antigens from gram-positive cocci. Cell. 1991;65: 1127–1141. 10.1016/0092-8674(91)90009-N 1905979

[28] Pizarro-Cerdá J, Kühbacher A, Cossart P. Entry of *Listeria monocytogenes* in mammalian epithelial cells: an updated view. Cold Spring Harb Perspect Med. 2012;2: 1–17. 10.1101/cshperspect.a010009 23125201 PMC3543101

[29] Cossart P, Vicente MF, Mengaud J, Baquero F, Perezdiaz JC, Berche P. Listeriolysin-O Is Essential for Virulence of *Listeria monocytogenes*—Direct Evidence Obtained By Gene Complementation. Infect Immun. 1989;57: 3629–3636. 2509366 10.1128/iai.57.11.3629-3636.1989 PMC259877

[30] Kocks C, Gouin E, Tabouret M, Berche P, Ohayon H, Cossart P. *L*. *monocytogenes*-induced actin assembly requires the actA gene product, a surface protein. Cell. 1992;68: 521–531. 10.1016/0092-8674(92)90188-I 1739966

[31] Leimeister-Wächter M, Domann E, Chakraborty T. Detection of a gene encoding a phosphatidylinositol-specific phospholipase C that is co-ordinately expressed with listeriolysin in *Listeria monocytogenes* . Mol Microbiol. 1991;5: 361–366. 1645838 10.1111/j.1365-2958.1991.tb02117.x

[32] Vazquezboland JA, Kocks C, Dramsi S, Ohayon H, Geoffroy C, Mengaud J, et al Nucleotide-Sequence of the Lecithinase Operon of *Listeria monocytogenes* and Possible Role of Lecithinase in Cell-To-Cell Spread. Infect Immun. 1992;60: 219–230. 1309513 10.1128/iai.60.1.219-230.1992 PMC257526

[33] Casino P, Rubio V, Marina A. The mechanism of signal transduction by two-component systems. Curr Opin Struct Biol. Elsevier Ltd; 2010;20: 763–771. 10.1016/j.sbi.2010.09.010 20951027

[34] Dasgupta A, Datta P, Kundu M, Basu J. The serine/threonine kinase PknB of *Mycobacterium tuberculosis* phosphorylates PBPA, a penicillin-binding protein required for cell division. Microbiology. 2006;152: 493–504. 10.1099/mic.0.28630-0 16436437

[35] Parikh A, Verma SK, Khan S, Prakash B, Nandicoori VK. PknB-mediated phosphorylation of a novel substrate, N-acetylglucosamine-1-phosphate uridyltransferase, modulates its acetyltransferase activity. J Mol Biol. Elsevier Ltd; 2009;386: 451–64. 10.1016/j.jmb.2008.12.031 19121323

[36] Kang C-M, Abbott DW, Park ST, Dascher CC, Cantley LC, Husson RN. The *Mycobacterium tuberculosis* serine/threonine kinases PknA and PknB: substrate identification and regulation of cell shape. Genes Dev. 2005;19: 1692–704. 10.1101/gad.1311105 15985609 PMC1176007

[37] Pietack N, Becher D, Schmidl SR, Saier MH, Hecker M, Commichau FM, et al In vitro phosphorylation of key metabolic enzymes from *Bacillus subtilis*: PrkC phosphorylates enzymes from different branches of basic metabolism. J Mol Microbiol Biotechnol. 2010;18: 129–40. 10.1159/000308512 20389117

[38] Liu Q, Fan J, Niu C, Wang D, Wang J, Wang X, et al The eukaryotic-type serine/threonine protein kinase stk is required for biofilm formation and virulence in *Staphylococcus epidermidis* . PLoS One. 2011;6: 1–13. 10.1371/journal.pone.0025380 21966513 PMC3179523

[39] Echenique J, Kadioglu A, Romao S, Andrew PW. Protein Serine / Threonine Kinase StkP Positively Controls Virulence and Competence in *Streptococcus pneumoniae*. 2004;72: 2434–2437. 10.1128/IAI.72.4.2434-2437.2004 PMC375209 15039376

[40] Ruggiero A, De Simone P, Smaldone G, Squeglia F, Berisio R. Bacterial cell division regulation by Ser/Thr kinases: a structural perspective. Curr Protein Pept Sci. 2012;13: 756–66. 23305362 10.2174/138920312804871201 PMC3601408

[41] Shah IM, Laaberki M-H, Popham DL, Dworkin J. A eukaryotic-like Ser/Thr kinase signals bacteria to exit dormancy in response to peptidoglycan fragments. Cell. Elsevier Inc.; 2008;135: 486–96. 10.1016/j.cell.2008.08.039 18984160 PMC2892110

[42] Fernandez P, Saint-Joanis B, Barilone N, Jackson M, Gicquel B, Cole ST, et al The Ser/Thr protein kinase PknB is essential for sustaining mycobacterial growth. J Bacteriol. 2006;188: 7778–7784. 10.1128/JB.00963-06 16980473 PMC1636329

[43] Wehenkel A, Fernandez P, Bellinzoni M, Catherinot V, Barilone N, Labesse G, et al The structure of PknB in complex with mitoxantrone, an ATP-competitive inhibitor, suggests a mode of protein kinase regulation in mycobacteria. FEBS Lett. 2006;580: 3018–3022. 10.1016/j.febslet.2006.04.046 16674948

[44] Lougheed KEA, Osborne SA, Saxty B, Whalley D, Chapman T, Bouloc N, et al Effective inhibitors of the essential kinase PknB and their potential as anti-mycobacterial agents. Tuberculosis (Edinb). Elsevier Ltd; 2011;91: 277–86. 10.1016/j.tube.2011.03.005 21482481 PMC3158675

[45] Bugrysheva J, Froehlich BJ, Freiberg JA, Scott JR. Serine/threonine protein kinase Stk is required for virulence, stress response, and penicillin tolerance in *Streptococcus pyogenes* . Infect Immun. 2011;79: 4201–4209. 10.1128/IAI.05360-11 21788381 PMC3187266

[46] Pensinger DA, Aliota MT, Schaenzer AJ, Boldon KM, Ansari IH, Vincent WJB, et al Selective pharmacologic inhibition of a PASTA kinase increases *Listeria monocytogenes* susceptibility to β-lactam antibiotics. Antimicrob Agents Chemother. 2014;58: 4486–94. 10.1128/AAC.02396-14 24867981 PMC4135996

[47] Foulquier E, Pompeo F, Freton C, Cordier B, Grangeasse C, Galinier A. PrkC-mediated phosphorylation of overexpressed YvcK protein regulates PBP1 protein localization in *Bacillus subtilis* mreB mutant cells. J Biol Chem. 2014;289: 23662–9. 10.1074/jbc.M114.562496 25012659 PMC4156092

[48] Görke B, Foulquier E, Galinier A. YvcK of *Bacillus subtilis* is required for a normal cell shape and for growth on Krebs cycle intermediates and substrates of the pentose phosphate pathway. Microbiology. 2005;151: 3777–91. 10.1099/mic.0.28172-0 16272399

[49] Foulquier E, Pompeo F, Bernadac A, Espinosa L, Galinier A. The YvcK protein is required for morphogenesis via localization of PBP1 under gluconeogenic growth conditions in *Bacillus subtilis* . Mol Microbiol. 2011;80: 309–18. 10.1111/j.1365-2958.2011.07587.x 21320184

[50] Mir M, Prisic S, Kang C-M, Lun S, Guo H, Murry JP, et al Mycobacterial gene cuvA is required for optimal nutrient utilization and virulence. Infect Immun. 2014;82: 4104–17. 10.1128/IAI.02207-14 25047842 PMC4187881

[51] Burke TP, Loukitcheva A, Zemansky J, Wheeler R, Boneca IG, Portnoy DA. *Listeria monocytogenes* Is Resistant to Lysozyme through the Regulation, Not the Acquisition, of Cell Wall-Modifying Enzymes. J Bacteriol. 2014;196: 3756–3767. 10.1128/JB.02053-14 25157076 PMC4248804

[52] Abachin E, Poyart C, Pellegrini E, Milohanic E, Fiedler F, Berche P, et al Formation of D-alanyl-lipoteichoic acid is required for adhesion and virulence of *Listeria monocytogenes* . Mol Microbiol. 2002;43: 1–14. 10.1046/j.1365-2958.2002.02723.x 11849532

[53] Joseph B, Przybilla K, Stühler C, Schauer K, Slaghuis J, Fuchs TM, et al Identification of *Listeria monocytogenes* Genes Contributing to Intracellular Replication by Expression Profiling and Mutant Screening. Society. 2006;188: 556–568. 10.1128/JB.188.2.556-568.2006 PMC1347271 16385046

[54] Eylert E, Schär J, Mertins S, Stoll R, Bacher A, Goebel W, et al Carbon metabolism of *Listeria monocytogenes* growing inside macrophages. Mol Microbiol. 2008;69: 1008–17. 10.1111/j.1365-2958.2008.06337.x 18627458

[55] Glomski IJ, Decatur AL, Portnoy DA. *Listeria monocytogenes* Mutants That Fail to Compartmentalize Listerolysin O Activity Are Cytotoxic, Avirulent, and Unable to Evade Host Extracellular Defenses. Infect Immun. 2003;71: 6754–6765. 10.1128/IAI.71.12.6754-6765.2003 14638761 PMC308949

[56] Rae CS, Geissler A, Adamson PC, Portnoy DA. Mutations of the *Listeria monocytogenes* peptidoglycan N-deacetylase and O-acetylase result in enhanced lysozyme sensitivity, bacteriolysis, and hyperinduction of innate immune pathways. Infect Immun. 2011;79: 3596–606. 10.1128/IAI.00077-11 21768286 PMC3165460

[57] Burke TP, Loukitcheva A, Zemansky J, Wheeler R, Boneca IG, Portnoy DA. *Listeria monocytogenes* Is Resistant to Lysozyme through the Regulation, Not the Acquisition, of Cell Wall-Modifying Enzymes. J Bacteriol. 2014;196: 3756–3767. 10.1128/JB.02053-14 25157076 PMC4248804

[58] Young TA, Delagoutte B, Endrizzi JA, Falick AM, Alber T. Structure of *Mycobacterium tuberculosis* PknB supports a universal activation mechanism for Ser/Thr protein kinases. Nat Struct Biol. 2003;10: 168–174. 10.1038/nsb897 12548283

[59] Bryant-Hudson KM, Shakir SM, Ballard JD. Autoregulatory characteristics of a *Bacillus anthracis* serine/threonine kinase. J Bacteriol. 2011;193: 1833–1842. 10.1128/JB.01401-10 21296958 PMC3133050

[60] Dephoure N, Gould KL, Gygi SP, Kellogg DR. Mapping and analysis of phosphorylation sites: a quick guide for cell biologists. Mol Biol Cell. 2013;24: 535–42. 10.1091/mbc.E12-09-0677 23447708 PMC3583658

[61] Kawashima S, Takemoto A, Nurse P, Kapoor T. A chemical biology strategy to analyze rheostat-like protein kinase-dependent regulation. Chem Biol. 2013;20: 262–271. 10.1016/j.chembiol.2013.01.003 23438755 PMC3626098

[62] Lima A, Durán R, Schujman GE, Marchissio MJ, Portela MM, Obal G, et al Serine/threonine protein kinase PrkA of the human pathogen *Listeria monocytogenes*: Biochemical characterization and identification of interacting partners through proteomic approaches. J Proteomics. Elsevier B.V.; 2011;74: 1720–1734. 10.1016/j.jprot.2011.03.005 21406257

[63] Macek B, Mijakovic I, Olsen JV, Gnad F, Kumar C, Jensen PR, et al The serine/threonine/tyrosine phosphoproteome of the model bacterium *Bacillus subtilis* . Mol Cell Proteomics. 2007;6: 697–707. 10.1074/mcp.M600464-MCP200 17218307

[64] Absalon C, Obuchowski M, Madec E, Delattre D, Holland IB, Séror SJ. CpgA, EF-Tu and the stressosome protein YezB are substrates of the Ser/Thr kinase/phosphatase couple, PrkC/PrpC, in *Bacillus subtilis* . Microbiology. 2009;155: 932–43. 10.1099/mic.0.022475-0 19246764

[65] Sajid A, Arora G, Gupta M, Singhal A, Chakraborty K, Nandicoori VK, et al Interaction of *Mycobacterium tuberculosis* elongation factor Tu with GTP is regulated by phosphorylation. J Bacteriol. 2011;193: 5347–5358. 10.1128/JB.05469-11 21803988 PMC3187401

[66] Nováková L, Sasková L, Pallová P, Janecek J, Novotná J, Ulrych A, et al Characterization of a eukaryotic type serine/threonine protein kinase and protein phosphatase of *Streptococcus pneumoniae* and identification of kinase substrates. FEBS J. 2005;272: 1243–54. 10.1111/j.1742-4658.2005.04560.x 15720398

[67] Misra SK, Moussan Désirée Aké F, Wu Z, Milohanic E, Cao TN, Cossart P, et al Quantitative Proteome Analyses Identify PrfA-Responsive Proteins and Phosphoproteins in *Listeria monocytogenes* . J Proteome Res. 2014;13: 6046–57. 10.1021/pr500929u 25383790

[68] Fleurie A, Manuse S, Zhao C, Campo N, Cluzel C, Lavergne J-P, et al Interplay of the serine/threonine-kinase StkP and the paralogs DivIVA and GpsB in pneumococcal cell elongation and division. PLoS Genet. 2014;10: e1004275 10.1371/journal.pgen.1004275 24722178 PMC3983041

[69] Kang C-M, Nyayapathy S, Lee J-Y, Suh J-W, Husson RN. Wag31, a homologue of the cell division protein DivIVA, regulates growth, morphology and polar cell wall synthesis in mycobacteria. Microbiology. 2008;154: 725–35. 10.1099/mic.0.2007/014076-0 18310019

[70] Giefing C, Jelencsics KE, Gelbmann D, Senn BM, Nagy E. The pneumococcal eukaryotic-type serine/threonine protein kinase StkP co-localizes with the cell division apparatus and interacts with FtsZ in vitro. Microbiology. 2010;156: 1697–1707. 10.1099/mic.0.036335-0 20223804

[71] Brundage RA, Smith GA, Camilli A, Theriot JA, Portnoy DA. Expression and phosphorylation of the *Listeria monocytogenes* ActA protein in mammalian cells. Proc Natl Acad Sci U S A. 1993;90: 11890–11894. 8265643 10.1073/pnas.90.24.11890 PMC48090

[72] Dworkin J. Ser/Thr phosphorylation as a regulatory mechanism in bacteria. Curr Opin Microbiol. Elsevier Ltd; 2015;24: 47–52. 10.1016/j.mib.2015.01.005 25625314 PMC4380854

[73] Beltramini AM, Mukhopadhyay CD, Pancholi V. Modulation of cell wall structure and antimicrobial susceptibility by a *Staphylococcus aureus* eukaryote-like serine/threonine kinase and phosphatase. Infect Immun. 2009;77: 1406–1416. 10.1128/IAI.01499-08 19188361 PMC2663143

[74] Tamber S, Schwartzman J, Cheung AL. Role of PknB kinase in antibiotic resistance and virulence in community-acquired methicillin-resistant *Staphylococcus aureus* strain USA300. Infect Immun. 2010;78: 3637–3646. 10.1128/IAI.00296-10 20547748 PMC2916262

[75] Liebeke M, Meyer H, Donat S, Ohlsen K, Lalk M. A metabolomic view of *Staphylococcus aureus* and its ser/thr kinase and phosphatase deletion mutants: involvement in cell wall biosynthesis. Chem Biol. Elsevier Ltd; 2010;17: 820–30. 10.1016/j.chembiol.2010.06.012 20797611

[76] Tomasz A. The mechanism of the irreversible antimicrobial effects of penicillins: how the beta-lactam antibiotics kill and lyse bacteria. Annu Rev Microbiol. 1979;33: 113–137. 10.1146/annurev.mi.33.100179.000553 40528

[77] Sauvage E, Kerff F, Terrak M, Ayala JA, Charlier P. The penicillin-binding proteins: structure and role in peptidoglycan biosynthesis. FEMS Microbiol Rev. 2008;32: 234–58. 10.1111/j.1574-6976.2008.00105.x 18266856

[78] Donat S, Streker K, Schirmeister T, Rakette S, Stehle T, Liebeke M, et al Transcriptome and functional analysis of the eukaryotic-type serine/threonine kinase PknB in *Staphylococcus aureus* . J Bacteriol. 2009;191: 4056–4069. 10.1128/JB.00117-09 19376851 PMC2698490

[79] Gardete S, Ludovice AM, Sobral RG, Filipe SR, De Lencastre H, Tomasz A. Role of murE in the Expression of β-Lactam Antibiotic Resistance in *Staphylococcus aureus* . J Bacteriol. 2004;186: 1705–1713. 14996801 10.1128/JB.186.6.1705-1713.2004 PMC355982

[80] Sieradzki K, Tomasz A. Suppression of β-lactam antibiotic resistance in a methicillin-resistant *Staphylococcus aureus* through synergic action of early cell wall inhibitors and some other antibiotics. J Antimicrob Chemother. 1997;39: 47–51. 10.1093/jac/39.suppl_1.47 9511062

[81] Sobral RG, Ludovice AM, Gardete S, Tabei K, De Lencastre H, Tomasz A. Normally functioning murF is essential for the optimal expression of methicillin resistance in *Staphylococcus aureus* . Microb Drug Resist. 2003;9: 231–241. 10.1089/107662903322286436 12959401

[82] Kawai Y, Daniel RA, Errington J. Regulation of cell wall morphogenesis in *Bacillus subtilis* by recruitment of PBP1 to the MreB helix. Mol Microbiol. 2009;71: 1131–44. 10.1111/j.1365-2958.2009.06601.x 19192185

[83] Cho H, Uehara T, Bernhardt TG. Beta-lactam antibiotics induce a lethal malfunctioning of the bacterial cell wall synthesis machinery. Cell. 2014;159: 1300–11. 10.1016/j.cell.2014.11.017 25480295 PMC4258230

[84] Price N, Tsvetanova B. Biosynthesis of the Tunicamycins: A Review. J Antibiot (Tokyo). 2007;60: 485–491. 17827659 10.1038/ja.2007.62

[85] Jani C, Eoh H, Lee JJ, Hamasha K, Sahana MB, Han J-S, et al Regulation of polar peptidoglycan biosynthesis by Wag31 phosphorylation in mycobacteria. BMC Microbiol. BioMed Central Ltd; 2010;10: 327 10.1186/1471-2180-10-327 21190553 PMC3019181

[86] Swoboda JG, Meredith TC, Campbell J, Brown S, Suzuki T, Bollenbach T, et al Discovery of a small molecule that blocks wall teichoic acid biosynthesis in *Staphlyococcus aureus* . ACS Chem Biol. 2010;4: 875–883. 10.1021/cb900151k. Discovery 19689117 PMC2787957

[87] Carvalho F, Atilano ML, Pombinho R, Covas G, Gallo RL, Filipe SR, et al L-Rhamnosylation of *Listeria monocytogenes* Wall Teichoic Acids Promotes Resistance to Antimicrobial Peptides by Delaying Interaction with the Membrane. PLOS Pathog. 2015;11: 1–29. 10.1371/journal.ppat.1004919 26001194 PMC4441387

[88] Chawla Y, Upadhyay S, Khan S, Nagarajan SN, Forti F, Nandicoori VK. Protein kinase B (PknB) of *Mycobacterium tuberculosis* is essential for growth of the pathogen in vitro as well as for survival within the host. J Biol Chem. 2014;289: 13858–13875. 10.1074/jbc.M114.563536 24706757 PMC4022859

[89] Débarbouillé M, Dramsi S, Dussurget O, Nahori MA, Vaganay E, Jouvion G, et al Characterization of a serine/threonine kinase involved in virulence of *Staphylococcus aureus* . J Bacteriol. 2009;191: 4070–4081. 10.1128/JB.01813-08 19395491 PMC2698471

[90] Gaidenko TA, Kim T, Price CW. The PrpC Serine-Threonine Phosphatase and PrkC Kinase Have Opposing Physiological Roles in Stationary-Phase *Bacillus subtilis* Cells J Bacteriol. 2002; 10.1128/JB.184.22.6109-6114.2002 PMC151969 12399479

[91] Archambaud C, Gouin E, Pizarro-Cerda J, Cossart P, Dussurget O. Translation elongation factor EF-Tu is a target for Stp, a serine-threonine phosphatase involved in virulence of *Listeria monocytogenes* . Mol Microbiol. 2005;56: 383–96. 10.1111/j.1365-2958.2005.04551.x 15813732

[92] Agirrezabala X, Frank J. Elongation in translation as a dynamic interaction among the ribosome, tRNA, and elongation factors EF-G and EF-Tu. Q Rev Biophys. 2009;42: 159–200. 10.1017/S0033583509990060 20025795 PMC2832932

[93] Russell JB, Cook GM. Energetics of bacterial growth: balance of anabolic and catabolic reactions. Microbiol Rev. 1995;59: 48–62. 7708012 10.1128/mr.59.1.48-62.1995 PMC239354

[94] Sanman LE, Qian Y, Eisele NA, Ng TM, van der Linden WA, Monack DM, et al Disruption of glycolytic flux is a signal for inflammasome signaling and pyroptotic cell death. Elife. 2016;5: e13663 10.7554/eLife.13663 27011353 PMC4846378

[95] Rajagopal L, Vo A, Silvestroni A, Rubens CE. Regulation of purine biosynthesis by a eukaryotic-type kinase in *Streptococcus agalactiae* . Mol Microbiol. 2005;56: 1329–1346. 10.1111/j.1365-2958.2005.04620.x 15882424 PMC2366208

[96] Forouhar F, Abashidze M, Xu H, Grochowski LL, Seetharaman J, Hussain M, et al Molecular insights into the biosynthesis of the F420 coenzyme. J Biol Chem. 2008;283: 11832–40. 10.1074/jbc.M710352200 18252724 PMC2431047

[97] Pilla DM, Hagar JA, Haldar AK, Mason AK, Degrandi D, Pfeffer K, et al Guanylate binding proteins promote caspase-11-dependent pyroptosis in response to cytoplasmic LPS. Proc Natl Acad Sci U S A. 2014;111: 6046–51. 10.1073/pnas.1321700111 24715728 PMC4000848

[98] Yamamoto M, Okuyama M, Ma JS, Kimura T, Kamiyama N, Saiga H, et al A cluster of interferon-γ-inducible p65 gtpases plays a critical role in host defense against *Toxoplasma gondii* . Immunity. 2012;37: 302–313. 10.1016/j.immuni.2012.06.009 22795875

[99] Horton RM, Cai ZL, Ho SN, Pease LR. Gene splicing by overlap extension: tailor-made genes using the polymerase chain reaction. Biotechniques. 1990;8: 528–35. Available: http://www.ncbi.nlm.nih.gov/pubmed/23573752357375 2357375

[100] Lauer P, Chow MYN, Loessner MJ, Portnoy DA, Calendar R. Construction, characterization, and use of two *Listeria monocytogenes* site-specific phage integration vectors. J Bacteriol. 2002;184: 4177–86. 10.1128/JB.184.15.4177-4186.2002 12107135 PMC135211

[101] Monk IR, Gahan CGM, Hill C. Tools for functional postgenomic analysis of *Listeria monocytogenes* . Appl Environ Microbiol. American Society for Microbiology (ASM); 2008;74: 3921–34. 10.1128/AEM.00314-08 18441118 PMC2446514

[102] Luu P, Gorman T. Short communication A chemically defined minimal medium of *Listeria* for the optimal culture. Int J Food Microbiol. 1997;35: 91–95. 9081230 10.1016/s0168-1605(96)01205-6

[103] Calderon-Miranda Marıa L, Barbosa-Canovas Gustavo V S BG. Transmission electron microscopy of *Listeria innocua* treated by pulsed electric fields and nisin in skimmed milk. Int J Food Microbiol. 1999;51: 31–38. 10.1016/S0168-1605(99)00071-9 10563461

[104] Jones S, Portnoy DA. Characterization of *Listeria monocytogenes* Pathogenesis in a Strain Expressing Perfringolysin O in Place of Listeriolysin O. Infect Immun. 1994;62: 5608–5613. 7960143 10.1128/iai.62.12.5608-5613.1994 PMC303309

[105] Blasi E, Mathieson BJ, Varesio L, Cleveland JL, Borchert PA, Rapp UR. Selective immortalization of murine macrophages from fresh bone marrow by a raf/myc recombinant murine retrovirus. Nature. 1985;318: 667–670. 10.1038/318667a0 4079980

[106] Decker T, Lohmann-Matthes ML. A quick and simple method for the quantitation of lactate dehydrogenase release in measurements of cellular cytotoxicity and tumor necrosis factor (TNF) activity. J Immunol Methods. 1988;115: 61–9. 3192948 10.1016/0022-1759(88)90310-9

