# Supplementary figures and images for: The Listeria monocytogenes PASTA Kinase PrkA and Its Substrate YvcK Are Required for Cell Wall Homeostasis, Metabolism, and Virulence
Source: PLoS Pathog. 2016 Nov 2;12(11):e1006001. doi: 10.1371/journal.ppat.1006001 (PMC5091766; doi:10.1371/journal.ppat.1006001)

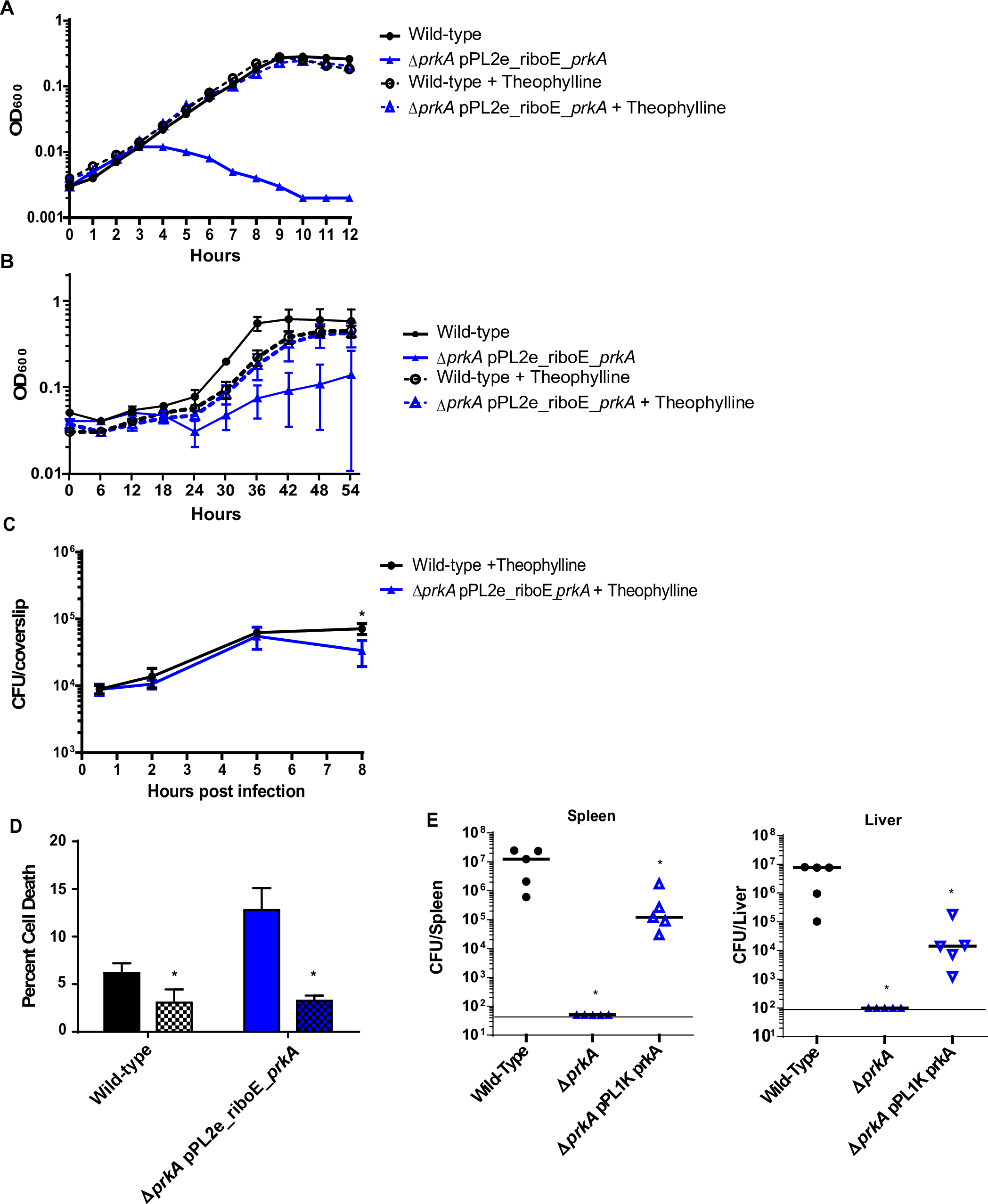

Supplement: S1 Fig — (A) Growth of wild-type (black circles) and the ΔprkA pPL2e_riboE_prkA mutant (blue triangles) in 0.0625μg/mL tunicamycin with or without 2mM Theophylline for complementation. Overnight cultures in BHI were grown in the absence (filled symbols and solid lines) or presence (open symbols and dashed lines) of 2mM Theophylline and back-diluted 1:50 into 96-well plates containing the same Theophylline concentration. Plates were grown at 37°C with continuous shaking for 12 hours in an Eon or Synergy HT Microplate Spectrophotometer (BioTek Instruments, Inc., Winooski, VT) and OD600 was read every hour. Growth curves are representative of 3 biological replicates. (B) Growth of wild-type (black circles) and the ΔprkA pPL2e_riboE_prkA mutant (blue triangles) in IMM Glycerol without (filled symbols and solid lines) or with 2mM Theophylline (open symbols and dashed lines) for complementation. Overnight BHI cultures were washed, inoculated into minimal media, grown at 37°C, and OD600 was measured every 6 hours. (C) Intracellular growth of wild-type (black circles) and ΔprkA mutants (blue triangles) was determined in bone marrow-derived macrophages (BMDMs) in media containing 1mM theophylline following infection at an MOI of 0.2. (D) Host cell death induced by wild-type (black) and ΔprkA pPL2e_riboE_prkA (blue) in uninduced (solid) or theophylline induced wells (checkered). BMDMs were infected with an MOI of 5 and complementation wells were maintained in 1mM Theophylline. Media supernatant was harvested at 6 hours and assayed for lactate dehydrogenase (LDH) activity from lysed macrophages. (E) C57Bl6 mice were infected intravenously with 1x10^5^ wild-type (black circles), ΔprkA mutants (blue triangles), or ΔprkA pPL1k prkA (empty blue triangles) in vivo. Spleens (left) and Livers (right) were harvested 48 hours post infection homogenized and plated for CFU. The median (solid bar) and limit of detection (line) for each experiment is indicated. Data are representative of two independ [file ppat.1006001.s001.tif]

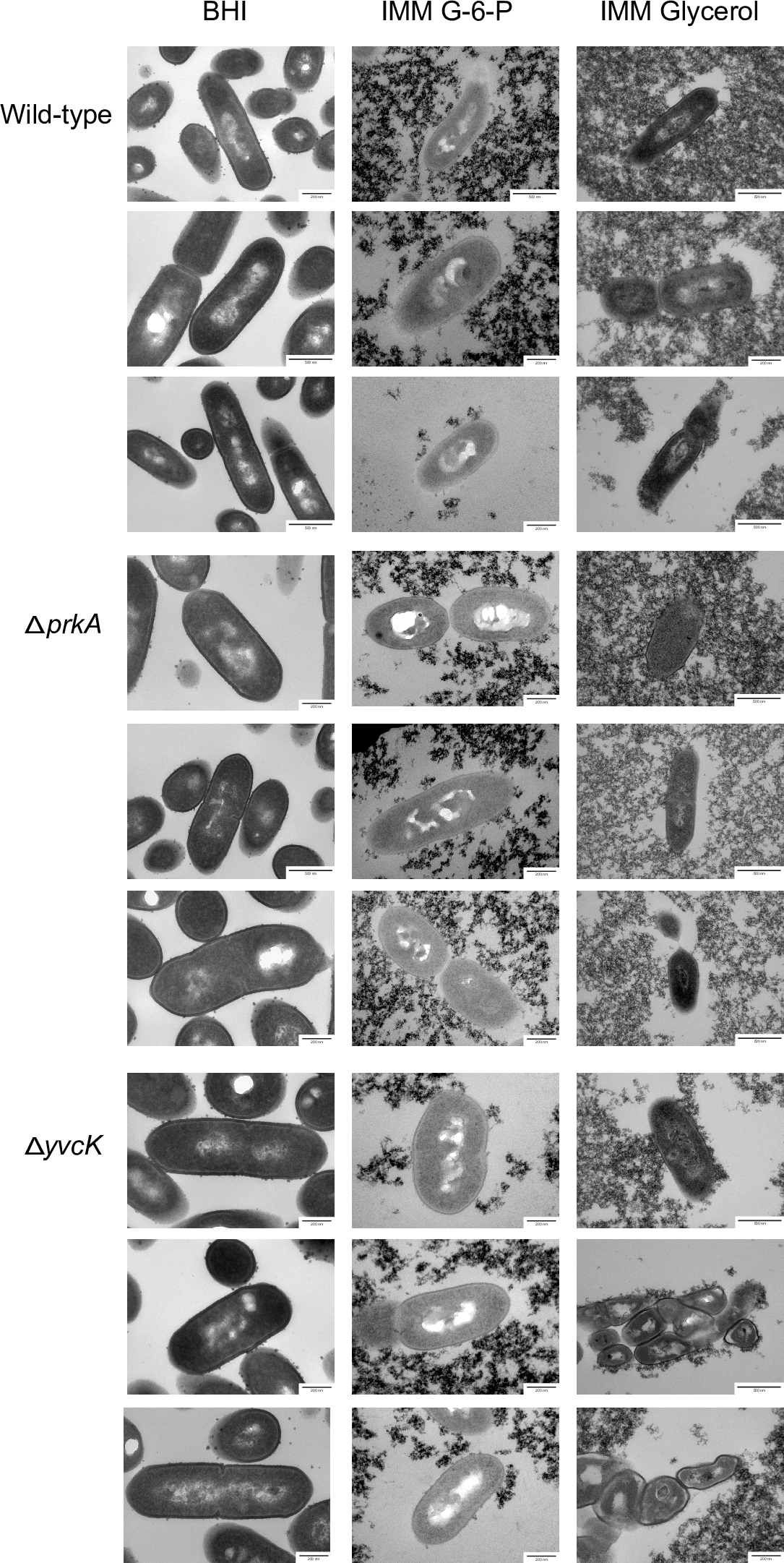

Supplement: S2 Fig — Morphology of wild-type, ΔprkA mutant, and ΔyvcK mutant in BHI, IMM Glucose-6-phosphate, and IMM Glycerol at OD 0.5 (BHI) or 6 hours post inoculation. (TIF) [file ppat.1006001.s002.tif]

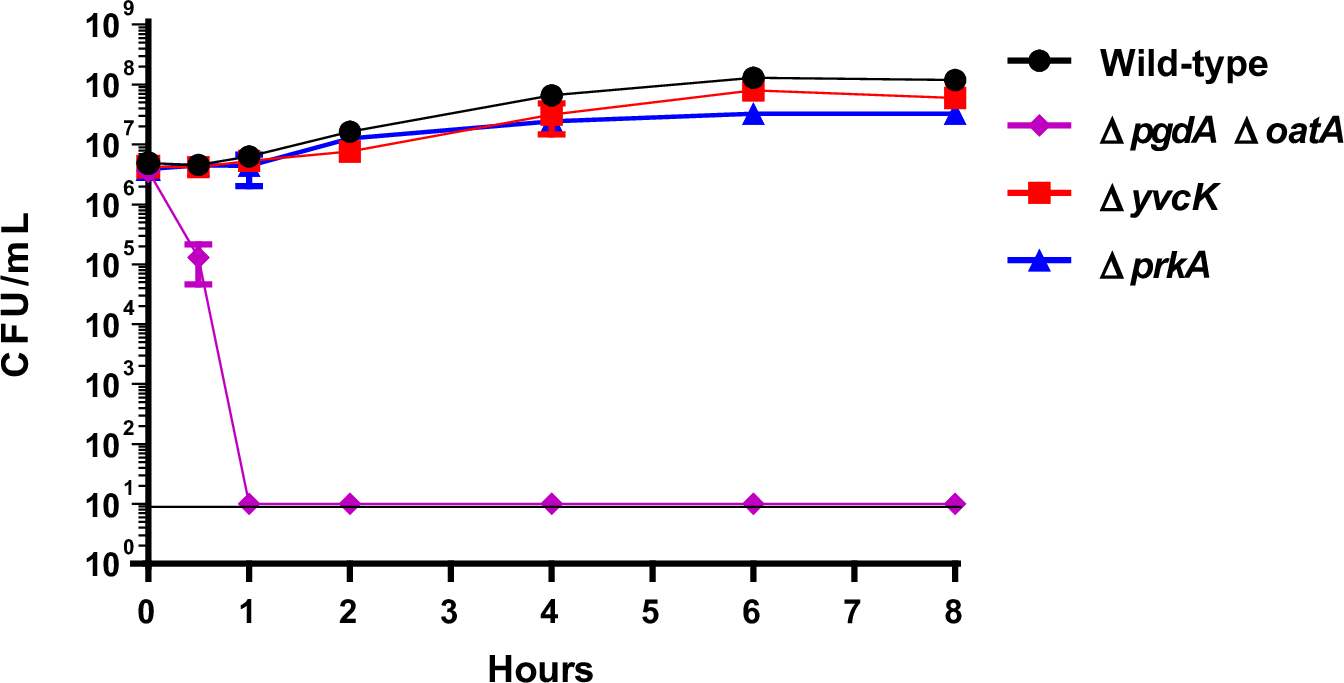

Supplement: S3 Fig — Survival of wild-type (black circles), ΔpgdA ΔoatA (purple diamonds), ΔyvcK (red squares), and ΔprkA (blue triangles) in defribronated sheep’s blood. Blood was inoculated with 5*106 CFU, incubated at 37°C, and plated for CFU at specified time points. Values are an average of 3 biological replicates and error bars represent standard deviation of the mean. (TIF) [file ppat.1006001.s003.tif]

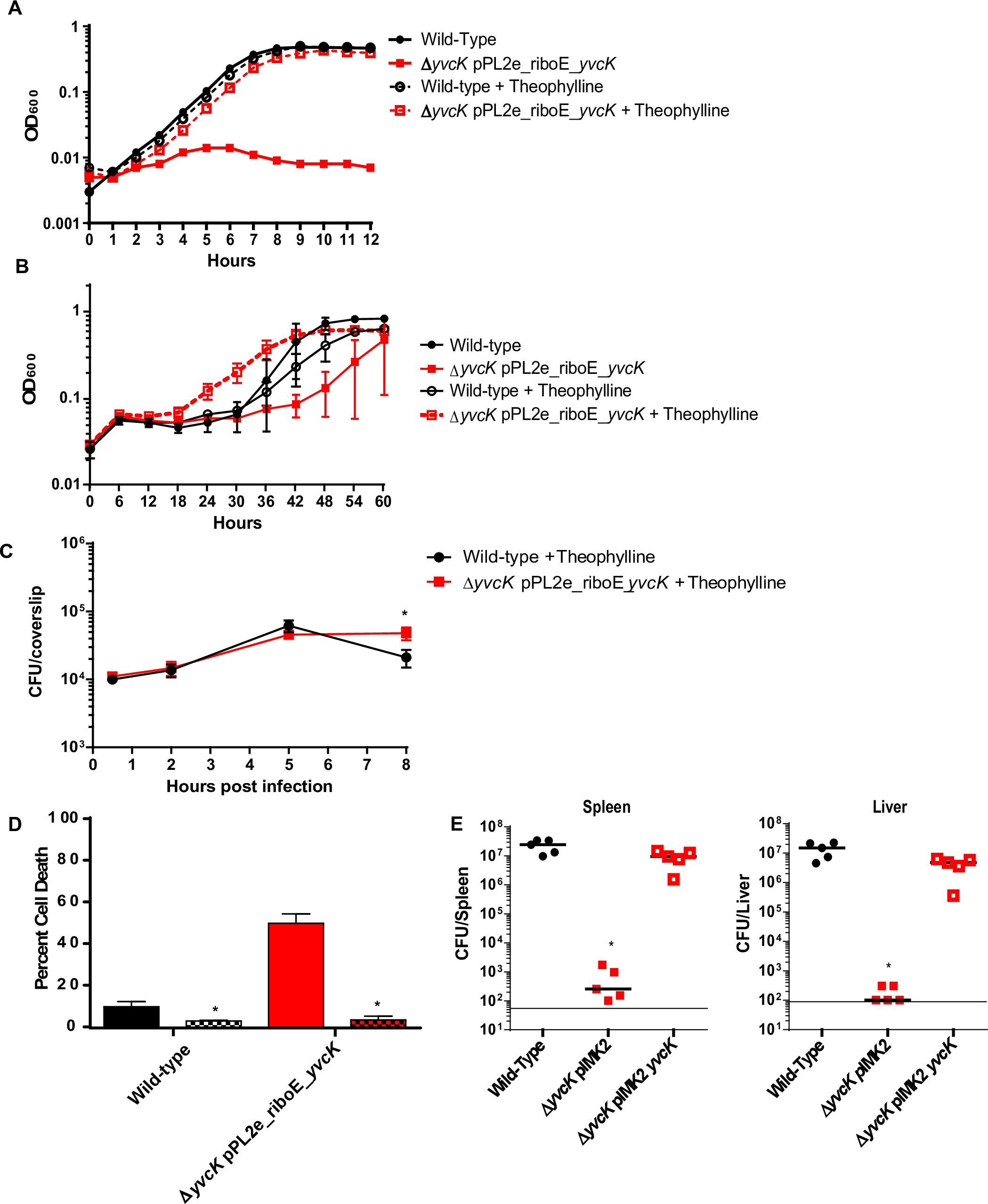

Supplement: S4 Fig — (A) Growth of wild-type (black circles) and the ΔyvcK pPL2e_riboE_yvcK mutant (red squares) in 1024μg/mL Lysozyme with or without 2mM Theophylline for complementation. Overnight cultures in BHI were grown in the absence (filled symbols and solid lines) or presence (open symbols and dashed lines) of 2mM Theophylline and back-diluted 1:50 into 96-well plates containing the same Theophylline concentration. Plates were grown at 37°C with continuous shaking for 12 hours in an Eon or Synergy HT Microplate Spectrophotometer (BioTek Instruments, Inc., Winooski, VT) and OD600 was read every hour. Growth curves are representative of 3 biological replicates. (B) Growth of wild-type (black circles) and the ΔyvcK pPL2e_riboE_yvcK mutant (red squares) in IMM Glycerol without (filled symbols and solid lines) or with 2mM Theophylline (open symbols and dashed lines) for complementation. Overnight BHI cultures were washed, inoculated into minimal media, grown at 37°C, and OD600 was measured every 6 hours. (C) Intracellular growth of wild-type (black circles) and ΔyvcK mutants (red squares) was determined in bone marrow-derived macrophages (BMDMs) in media containing 1mM theophylline following infection at an MOI of 0.2. (D) Host cell death induced by wild-type (black) and ΔyvcK pPL2e_riboE_yvcK (red) in uninduced (solid) or theophylline induced wells (checkered). BMDMs were infected with an MOI of 5 and complementation wells were maintained in 1mM Theophylline. Media supernatant was harvested at 6 hours and assayed for lactate dehydrogenase (LDH) activity from lysed macrophages. (E) C57Bl6 mice were infected intravenously with 1x10^5^ wild-type (black circles), ΔyvcK pIMK2 empty (red squares), or ΔyvcK pIMK2 yvcK (empty red squares) in vivo. Spleens (left) and Livers (right) were harvested 48 hours post infection homogenized and plated for CFU. The median (solid bar) and limit of detection (line) for each experiment is indicated. Data are representative of two independent experiments w [file ppat.1006001.s004.tif]

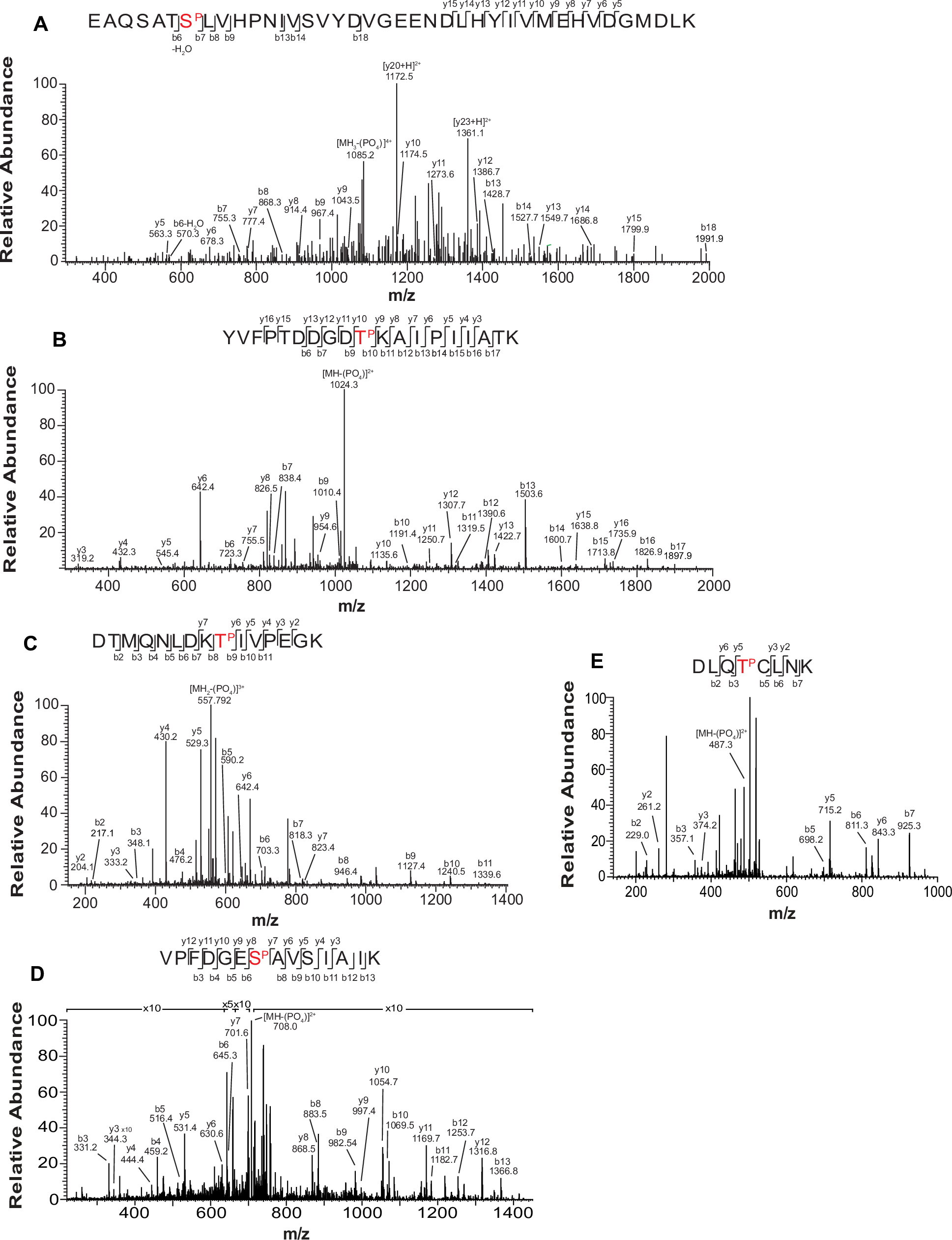

Supplement: S5 Fig — MS/MS spectra of PrkA phosphopeptides. S64 (A), T289 (B), and T307 (C) were identified as autophosphorylation sites from three separate tryptic peptides. S64 phosphorylation in peptide 58–96 can be identified definitively solely through b ions. T289 phosphorylation in peptide 280–298 can be identified definitively through the combination of b and y ions. T307 phosphorylation in peptide 299–313 can be identified definitively by either b or y ions. Phosphopeptides identified post titanium oxide enrichment identified S213 (D) and T268 (E) as autophosphorylation sites. S213 phosphorylation and T268 phosphorylation in peptides 207–220 and 265–272 can be identified definitively by either b or y ions. (TIF) [file ppat.1006001.s005.tif]
